# Meta‐Analysis: Redefining Liver Disease Risk in Heterozygous Alpha‐1 Antitrypsin Deficiency

**DOI:** 10.1111/apt.70814

**Published:** 2026-07-12

**Authors:** Adam M. Syanda, Dimitra Georgantaki, Riona T. T. Linn, Amara Agbai, Hassan Tahir, Manako Sakai, Richard Thompson, Mariam Molokhia, S. Tamir Rashid

**Affiliations:** ^1^ Department of Metabolism, Digestion and Reproduction, Faculty of Medicine Imperial College London London UK; ^2^ Department of Population Health Sciences School of Life Course and Population Sciences, King's College London London UK; ^3^ Faculty of Medicine Imperial College London London UK; ^4^ Institute of Liver Studies King's College London London UK

**Keywords:** A1ATD, alpha‐1 antitrypsin deficiency, heterozygous MZ, heterozygous SZ, liver cirrhosis, liver disease, liver fibrosis, meta‐analysis, SERPINA1, steatosis

## Abstract

**Background:**

Heterozygous SERPINA1 genotypes (MZ, SZ) have historically been regarded as carrier states with limited liver relevance. Emerging evidence suggests increased liver injury, but the magnitude of risk remains uncertain. We synthesized evidence on heterozygous SERPINA1 liver phenotypes to quantify this risk.

**Methods:**

We performed a systematic review of bibliographic databases from inception to 21 October 2025 for studies reporting liver outcomes in MZ or SZ genotypes. Outcomes were grouped into metabolic comorbidities, biochemical markers, and clinical liver disease. Random‐effects meta‐analyses pooled mean differences (MDs) and odds ratios (ORs); risk of bias was assessed with established tools (PROSPERO CRD420251165586).

**Results:**

Twenty‐six studies including 27,933 heterozygotes and 547,961 controls met inclusion criteria. BMI (MD −0.09 [−0.20–0.02]) and steatosis risk (OR 1.09 [0.74–1.60]) were comparable between groups. Heterozygotes had modestly higher liver enzymes (ALT MD 1.03 [0.41–1.66]; AST MD 0.68 [0.51–0.85]; ALP MD 2.58 [1.58–3.58]) and higher odds of abnormal ALT (OR 1.18 [1.11–1.25]), AST (OR 1.16 [1.07–1.26]), and ALP (OR 1.41 [1.18–1.68]). They had significantly increased odds of fibrosis (OR 2.14 [1.52–3.02]), cirrhosis (OR 2.24 [1.73–2.91]), and liver transplantation (OR 2.28 [1.45–3.59]).

**Conclusions:**

Heterozygous SERPINA1 genotypes are bona fide liver risk states, with effect sizes comparable to established genetic and environmental determinants. MZ/SZ heterozygosity should be recognized as moderate‐penetrance liver susceptibility genotypes in chronic liver disease risk assessment.

## Graphical abstract

SERPINA1 MZ/SZ genotypes is a common but under‐recognised liver risk state. Despite modest shifts in liver function tests, it is associated with approximately doubled odds of fibrosis, cirrhosis, and liver transplantation, supporting its inclusion in chronic liver disease risk assessment.

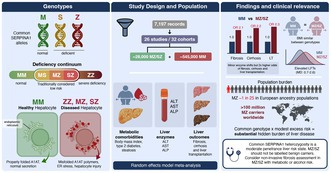

**Abbreviations**
A1AT: alpha‐1 antitrypsin
A1ATD: alpha‐1 antitrypsin deficiency
ALP: alkaline phosphatase
ALT: alanine aminotransferase
AST: aspartate aminotransferase
BMI: body mass index
CI: confidence interval
ER: endoplasmic reticulum
iPSC: induced pluripotent stem cell
kPa: kilopascal
MD: mean difference
MM: wild‐type SERPINA1 genotype (PiMM)
MZ: heterozygous SERPINA1 genotype (PiMZ)
NOS: Newcastle–Ottawa Scale
OR: odds ratio
PNPLA3: patatin‐like phospholipase domain‐containing protein 3
PRISMA: Preferred Reporting Items for Systematic Reviews and Meta‐Analyses
PROSPERO: International Prospective Register of Systematic Reviews
SZ: heterozygous SERPINA1 genotype (PiSZ)
TM6SF2: transmembrane 6 superfamily member 2
UK: United Kingdom

## 1 Introduction

Alpha‐1 antitrypsin deficiency (A1ATD) is an autosomal codominant disorder caused by pathogenic variants in the SERPINA1 gene. Mutations in this gene can reduce the amount and impair the function of alpha‐1 antitrypsin (A1AT), predisposing affected individuals to progressive lung and liver disease [1, 2, 3, 4, 5, 6]. In individuals with the wild‐type M allele, A1AT is an acute‐phase glycoprotein synthesized by hepatocytes, released into the bloodstream, and delivered to the lungs where it limits neutrophil elastase activity [7, 8]. When A1AT is deficient or dysfunctional due to misfolding, neutrophil‐derived proteases cause cumulative structural damage within the airways and pulmonary interstitium, leading to the early onset of emphysema [9, 10]. In parallel, hepatocellular retention of misfolded A1AT within the endoplasmic reticulum (ER) has been shown to drive unfolded protein response [11], inflammation [12] and progressive fibrosis [13], establishing the liver as a primary site of injury.

The pathogenic Z allele (rs28929474) arises from a single nucleotide substitution in SERPINA1 (c.1096G> A) that replaces glutamic acid with lysine at position 342 (Glu342Lys) in the A1AT polypeptide [14, 15, 16]. This amino acid change destabilizes protein folding, such that only a small proportion of Z‐A1AT is secreted while the majority polymerizes and is retained within the hepatocyte endoplasmic reticulum (ER), driving cellular stress, inflammation, cell death, and progressive fibrosis [17]. The S allele is defined by a missense variant that substitutes glutamic acid at codon 264 with valine (Glu264Val), resulting in a protein that is secreted albeit at reduced levels but far less prone to polymerization than the Z‐A1AT equivalent [6, 18]. In combination with the wild‐type M allele, these variants generate a continuum of SERPINA1 genotypes—from severe deficiency states (ZZ) to heterozygous forms (MZ, MS, SZ, FM, IM, and others) that give rise to a broad spectrum of clinical phenotypes.

Current clinical paradigms largely accept that individuals homozygous for the Z allele are at substantial risk of liver disease [19], with a bimodal age distribution spanning neonatal jaundice and adult cirrhosis [19, 20, 21]. In contrast, heterozygous states—in particular the common MZ genotype—have historically been labelled as benign carriers [22, 23], or at most as susceptible only in the presence of additional hepatic insults. This reassuring narrative sits uneasily with several biological and epidemiological signals. Population estimates suggest that heterozygous pathogenic genotypes are extremely common worldwide, with up to 185 million individuals harboring the Z allele [24]. Even a modest increase in liver risk at the individual level will therefore translate into a substantial, and currently unrecognized, burden of disease for hundreds of thousands of individuals at the population level.

More recently, several observational studies have challenged the notion that MZ heterozygosity is clinically irrelevant [25, 26, 27]. MZ individuals have been reported to be over‐represented among patients with cirrhosis, decompensated liver disease and those listed for liver transplantation, particularly in the presence of common cofactors such as alcohol use, metabolic dysfunction‐associated steatotic liver disease and chronic inflammatory or degenerative conditions [27, 28]. Other studies, often in smaller or differently ascertained cohorts, have failed to demonstrate a clear excess risk of advanced liver disease among MZ carriers compared with wild‐type MM controls [22, 23]. Taken together, the available evidence suggests that MZ may confer increased susceptibility to progressive liver injury, particularly in the presence of metabolic or environmental stressors, although the magnitude of risk and the contribution of confounding remain uncertain [22]. This uncertainty has immediate implications for clinical practice and policy. Existing guidelines do not clearly define whether MZ status alone should prompt targeted surveillance for chronic liver disease or structured counseling on lifestyle and metabolic comorbidities, and recommendations remain inconsistent across healthcare systems. In routine practice, MZ carriers are still largely regarded as low risk, despite accumulating but fragmented evidence suggesting a higher burden of liver disease in this group.

In this context, a rigorous synthesis of the available data is urgently needed. The objective of this systematic review and meta‐analysis was to collate and critically appraise all published evidence on the relationship between heterozygous MZ and SZ A1ATD genotypes, and the prevalence, severity, and clinical outcomes of liver disease compared with wild‐type homozygous cohorts. By quantifying the magnitude and consistency of any association across diverse settings, this review aims to clarify whether SERPINA1 heterozygosity should be considered a genuine liver risk state and to inform future screening, monitoring, and policy strategies for this large and currently under‐recognized population.

## 2 Methods

### 2.1 Search Strategy and Selection Criteria

A comprehensive systematic search was conducted to identify studies reporting liver outcomes in individuals heterozygous for MZ or SZ alpha‐1 antitrypsin deficiency (A1ATD) (Table S1). Electronic databases were searched from inception to 21 October 2025, including Ovid (MEDLINE, MEDLINE In‐Process, EMBASE, Global Health, Journals@Ovid), EBSCO (CINAHL), PubMed Central, the Cochrane Library (Cochrane Reviews, Cochrane Protocols, Cochrane Central Register of Controlled Trials), EU Clinical Trials Register, NIHR Health Technology Assessment, NHS Economic Evaluation Database, Database of Abstracts of Reviews of Effects, Web of Science (Clarivate), ClinicalTrials.gov, ISRCTN Registry, WHO International Clinical Trials Registry Platform, and medRxiv (preprints).

Eligible studies included human participants with confirmed SERPINA1 genotype and reported data for MZ and/or SZ heterozygotes, with extractable information on liver‐related outcomes and, where available, comparator groups (MM genotype or general population). Exclusion criteria comprised animal or cell‐based studies, publications focused exclusively on homozygous A1ATD or genotypes outside the predefined heterozygous variants (MZ and SZ), and non‐English language publications. After manual and semi‐automated deduplication, titles and abstracts were screened in Rayyan [29] by six reviewers working in three independent pairs. Publications were divided equally between pairs, with both reviewers in each pair independently screening the same allocated records. Disagreements were resolved by discussion within the review team, with input from senior clinical hepatology consultants where required. Full texts of potentially eligible articles were then assessed for extractable data on MZ and SZ heterozygotes and their matched or otherwise defined controls. The systematic review and meta‐analysis were reported in accordance with PRISMA guidelines, with study selection, design, and findings documented in the PRISMA checklist (Table S3). The systematic review was registered on PROSPERO: prospective register of systematic reviews (CRD420251165586).

Eligible study designs comprised case series with ≥ 5 heterozygous participants, cross‐sectional studies, case–control studies, and cohort studies. Studies without MM or general population comparators were eligible for prevalence estimation but were excluded from comparative meta‐analyses. However, these studies were excluded from comparative meta‐analyses if participants were recruited specifically on the basis of liver outcomes. Quantitative synthesis of crude odds ratios (ORs) and mean differences (MDs) was restricted to studies that reported outcomes for MZ or SZ heterozygotes alongside MM (wild‐type) or general population comparators.

### 2.2 Data Extraction and Quality Assessment

Data were extracted by six reviewers working in three independent pairs, using a piloted, standardized extraction form. Within each pair, both reviewers extracted data from the same allocated publications and discrepancies were resolved by consensus, with arbitration by a senior hepatology reviewer where required. Potential overlap between publications drawing on the same underlying cohorts was assessed by comparing study setting, recruitment period, population characteristics, and sample descriptors. Where overlap was likely, the most recent study reporting the relevant outcomes was included; where the more recent study did not report all outcomes of interest, the more complete study was selected.

Study quality was assessed using the Newcastle‐Ottawa Scale, covering selection, comparability, and outcome or exposure assessment domains (Figure S2). Studies were appraised independently in duplicate, with disagreements resolved by consensus, and assigned an overall low, moderate, or high risk‐of‐bias classification according to prespecified criteria. All eligible studies were retained in the meta‐analyses.

### 2.3 Data Analysis

Analyses were structured a priori into three domains. First, we evaluated metabolic comorbidities, including obesity, diabetes, and hepatic steatosis. Obesity was defined by body mass index (BMI) ≥ 30 kg/m^2^, and type 2 diabetes mellitus prevalence was determined from clinically diagnosed cases reported in the included studies. Steatosis, defined as excessive hepatic fat accumulation, was ascertained from histology or from controlled attenuation parameter consistent with S2 score or higher. Second, we analyzed biochemical markers of liver injury: alanine aminotransferase (ALT), aspartate aminotransferase (AST), and alkaline phosphatase (ALP). The prevalence of elevated liver enzymes was calculated from the reported proportion of individuals whose values exceeded sex‐specific and age‐appropriate clinical reference limits. Third, we assessed clinical liver outcomes, including fibrosis, cirrhosis, and liver transplantation. Fibrosis was defined by transient elastography with liver stiffness > 7.1 kPa or by histology showing bridging fibrosis or worse (METAVIR ≥ F2). Cirrhosis was based on histological staging (METAVIR ≥ F4) and/or clinical diagnosis. Liver transplantation was defined as being listed for, or having received, a liver transplant.

Random‐effects meta‐analyses using the DerSimonian‐Laird estimator were applied throughout. Prevalence estimates were analyzed on the logit scale using inverse‐variance weighting and back‐transformed to percentages for presentation. Continuous outcomes were pooled as mean differences (MDs). Binary comparative outcomes were analyzed as odds ratios (ORs), calculated from 2 × 2 contingency tables and pooled on the log scale before back‐transformation. Between‐study heterogeneity was quantified using Cochran's Q, τ^2^, and *I* ^2^. *I* ^2^ values of approximately 25%, 50%, and 75% were interpreted as low, moderate, and high heterogeneity, respectively, although these thresholds were used as guides rather than absolute cut‐offs.

For each meta‐analyzed outcome, we performed sensitivity analyses to assess the robustness of the pooled estimates. Leave‐one‐out analyses were used to evaluate the influence of individual studies by repeating each meta‐analysis after sequentially omitting one study at a time. Subgroup analyses were then performed to explore whether pooled estimates varied by SERPINA1 genotype and age group. Genotype‐stratified analyses were conducted for MZ, SZ, and combined heterozygous groups where data were available. Age‐stratified analyses compared pediatric and adult cohorts, restricted to outcomes for which both age groups were reported.

Subgroup differences were assessed using mixed‐effects meta‐regression, with subgroup entered as a categorical moderator. Models were fitted by inverse‐variance weighted least squares, with residual between‐study heterogeneity estimated using the DerSimonian‐Laird method‐of‐moments estimator. For two‐level subgroup comparisons, the moderator coefficient was tested using a Wald z test. For categorical moderators with more than two levels, overall subgroup differences were assessed using an omnibus Wald chi‐square test of moderators, reported as the QM statistic with degrees of freedom equal to the number of non‐reference subgroup levels.

Publication bias and small‐study effects were assessed using funnel plots and Egger regression on the appropriate model scale: mean difference for continuous outcomes, log odds ratio for binary comparative outcomes, and logit‐transformed prevalence for prevalence meta‐analyses. Egger regression modeled the standardized effect estimate against study precision, with intercept deviation from zero used as evidence of funnel plot asymmetry. Analyses were performed in Python using custom scripts based on NumPy, SciPy, math, and statsmodels packages.

## 3 Results

### 3.1 Study Selection and Characteristics

From 7394 records identified through literature database searches (Table S1), 26 studies met the eligibility criteria and were included in the review (Table S2 and Figure S1A). These studies contributed 32 distinct MZ or SZ heterozygous cohorts, comprising 27,933 individuals. The earliest included study was published in 1985, although most evidence came from the past decade: two studies were published before 2006, six between 2006 and 2015, and 18 between 2016 and 2025 (69.2%; Figure S1B).

The aggregated heterozygous dataset comprised 21,212 MZ individuals (75.9%), 807 SZ individuals (2.9%), and 5914 mixed MZ/SZ heterozygotes (21.2%), with 547,961 matched MM or population comparators. Sex distribution was broadly balanced across genotype groups, with no significant difference between groups (*p* = 0.576; Figure S1C). The evidence base was geographically concentrated, with the largest contributions from the United States, United Kingdom, Germany, and Sweden (Figure S1D). Study‐quality assessment using the Newcastle‐Ottawa Scale rated 85% of studies as fair quality and 15% as high quality; no low‐quality studies were included (Figure S2). Follow‐up completeness and participant attrition were the main contributors to lower quality scores. Sensitivity analyses were performed using leave‐one‐out analysis and subgroup stratification by genotype and age group (Figure S3–S7). No significant differences between subgroups were found for all reported outcomes. Publication bias and small‐study effects were assessed using funnel plots and Egger regression. There was no evidence of funnel plot asymmetry across all outcomes (Figure S8).

### 3.2 Metabolic Comorbidities: Obesity, Type 2 Diabetes Mellitus and Steatosis

**FIGURE 1:**
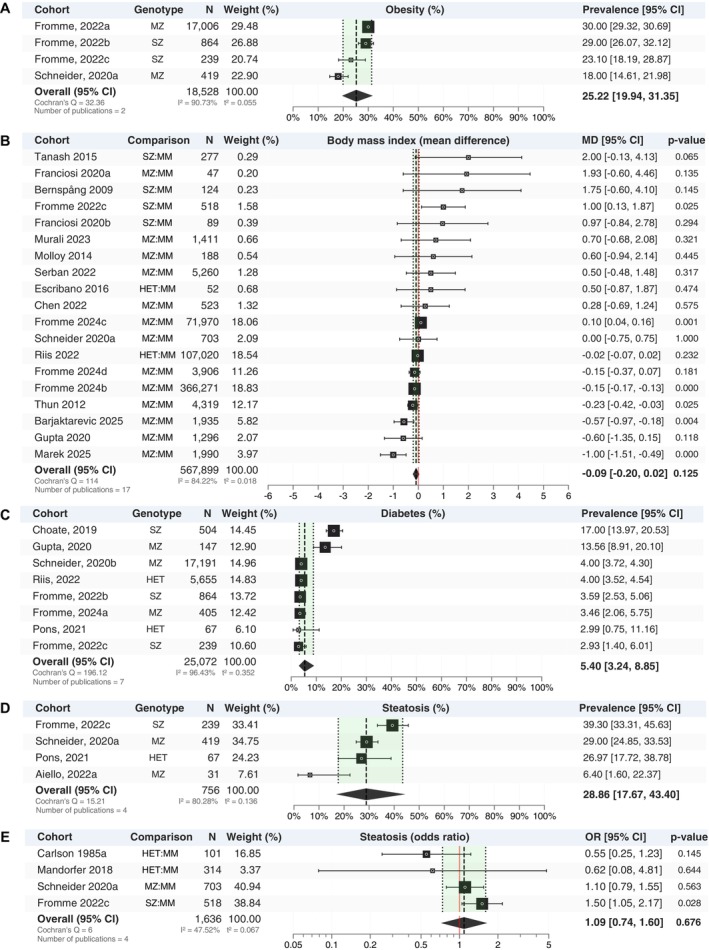
Metabolic comorbidities in heterozygous SERPINA1 genotypes. Forest plots show pooled estimates for obesity prevalence (A), BMI mean difference versus MM controls (B), type 2 diabetes prevalence (C), steatosis prevalence (D), and steatosis odds ratio versus MM controls (E) among individuals with MZ, SZ, or combined heterozygous SERPINA1 genotypes. Green area represents pooled 95% CI. CI = confidence interval; HET = heterozygous; MD = mean difference; OR = odds ratio.

The metabolic comorbidities, obesity and type 2 diabetes mellitus, are known risk factors of liver disease [30, 31, 32]. Across adult heterozygotes, the pooled prevalence of obesity was 25.2% (95% CI 19.9–31.4; *I* ^2^ = 90.7%; Figure 1A). BMI did not differ between heterozygotes and MM controls (MD −0.09, 95% CI −0.20 to 0.02; *p* = 0.125; *I* ^2^ = 84.2%; Figure 1B).

The pooled prevalence of type 2 diabetes was 5.4% (95% CI 3.24–8.85; *I* ^2^ = 96.4%; Figure 1C). Hepatic steatosis was present in approximately one third of heterozygotes, with a pooled prevalence of 28.9% (95% CI 17.67–43.40; *I* ^2^ = 80.3%; Figure 1D). In comparative analyses, steatosis odds did not differ clearly between heterozygotes and MM controls (OR 1.09, 95% CI 0.74–1.60; *p* = 0.68; *I* ^2^ = 47.5%; Figure 1E) and were in line with what has been reported in the general population [33].

### 3.3 Biochemical Markers of Liver Injury

**FIGURE 2:**
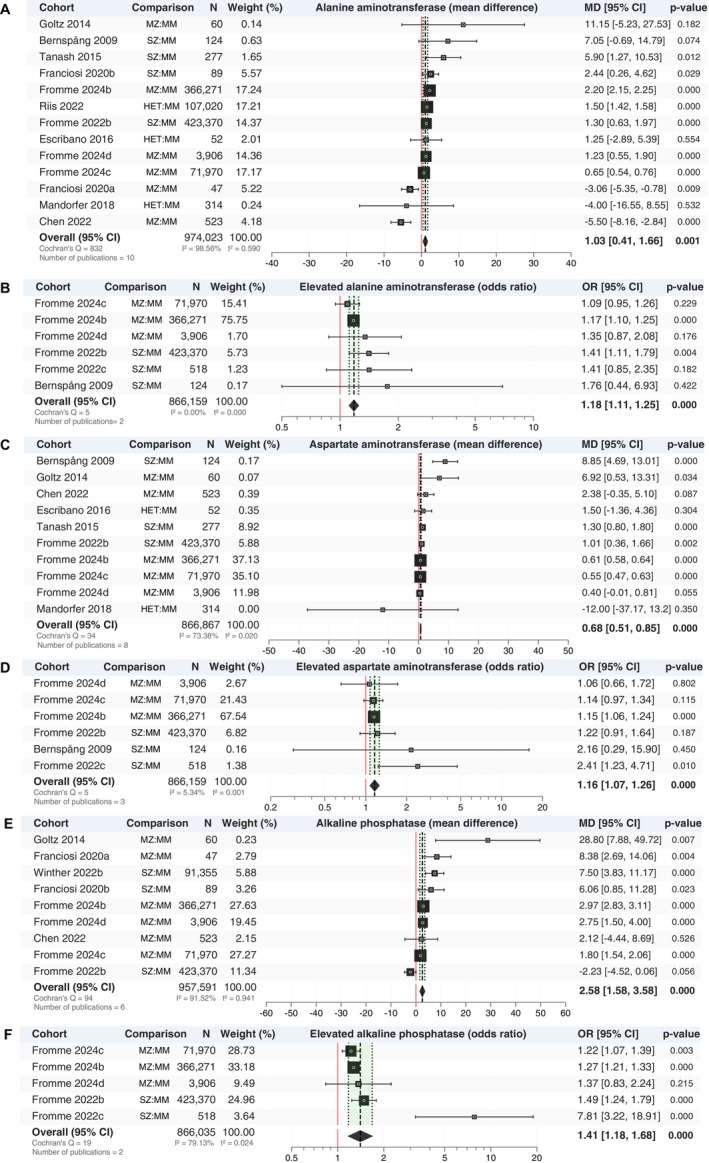
Liver biochemistry in heterozygous SERPINA1 genotypes. Forest plots show pooled mean differences for alanine aminotransferase (ALT) (A), aspartate aminotransferase (AST) (C), and alkaline phosphatase (ALP) (E), and pooled odds ratios for elevated ALT (B), elevated AST (D), and elevated ALP (F), in individuals with MZ, SZ, or combined heterozygous SERPINA1 genotypes. Green area represents pooled 95% CI. CI = confidence interval; HET = heterozygous; MD = mean difference; OR = odds ratio.

We next examined whether heterozygous A1ATD was associated with biochemical evidence of liver injury on routine liver function testing. Heterozygous SERPINA1 genotypes were associated with higher liver enzyme concentrations than MM genotypes. ALT was higher in heterozygotes than in MM controls (MD 1.03, 95% CI 0.41–1.66; *p* = 0.001; *I* ^2^ = 98.6%; Figure 2A), and the odds of ALT elevation were increased (OR 1.18, 95% CI 1.11–1.25; *p* < 0.0001; *I* ^2^ = 0.0%; Figure 2B). AST showed a similar pattern, with higher concentrations in heterozygotes (MD 0.68, 95% CI 0.51–0.85; *p* < 0.0001; *I* ^2^ = 73.3%; Figure 2C) and increased odds of elevation (OR 1.16, 95% CI 1.07–1.26; p < 0.0001; 866,159 participants; *I* ^2^ = 5.3%; Figure 2D).

ALP concentrations were also higher in heterozygotes than in MM controls (MD 2.58, 95% CI 1.58–3.58; p < 0.0001; *I* ^2^ = 91.5%; Figure 2E), with increased odds of values above the upper limit of normal (OR 1.41, 95% CI 1.18–1.68; *p* < 0.0001; *I* ^2^ = 79.1%; Figure 2F). Overall, biochemical differences were small in absolute magnitude but directionally consistent across liver enzyme outcomes.

### 3.4 Liver Disease Outcomes: Fibrosis, Cirrhosis and Liver Transplantation

**FIGURE 3:**
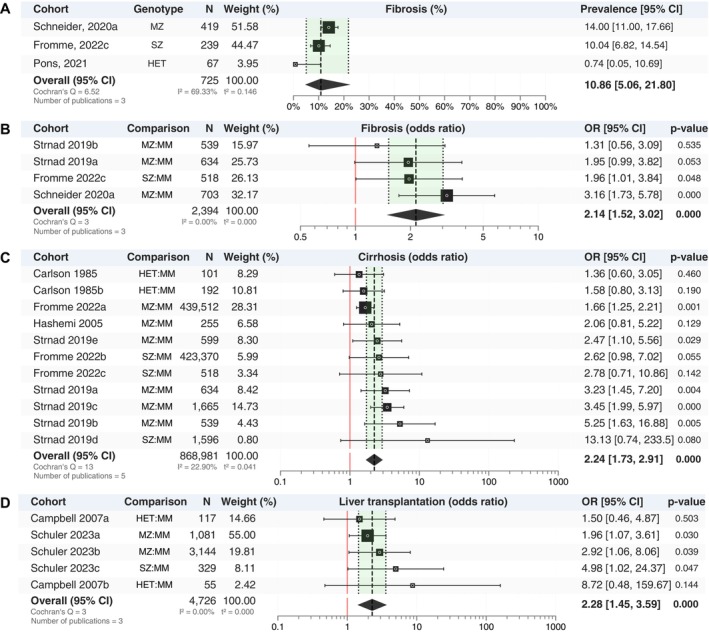
Fibrosis, cirrhosis, and liver transplantation in heterozygous SERPINA1 genotypes. Pooled estimates are presented for fibrosis prevalence (A), fibrosis odds ratio versus MM controls (B), cirrhosis odds ratio versus MM controls (C), and liver transplantation odds ratio versus MM controls in MZ, SZ, and combined heterozygous SERPINA1 genotypes (D). Heterozygotes had increased odds of fibrosis, cirrhosis, and liver transplantation compared with MM controls. CI = confidence interval; HET = heterozygous; OR = odds ratio.

Significant fibrosis, assessed by histology or transient elastography, was present in approximately one in ten heterozygotes (pooled prevalence 10.86%, 95% CI 5.06–21.80; 725 adults; *I* ^2^ = 69.3%; Figure 3A). In comparative analyses, heterozygotes had more than two‐fold higher odds of fibrosis than MM controls (OR 2.14, 95% CI 1.52–3.02; *p* < 0.0001; *I* ^2^ = 0.0%; Figure 3B).

Heterozygotes also had increased odds of cirrhosis compared with MM controls (OR 2.24, 95% CI 1.73–2.91; p < 0.0001; *I* ^2^ = 22.9%; Figure 3 C). Similarly, the odds of being listed for, or receiving, a liver transplant were increased in heterozygotes (OR 2.28, 95% CI 1.45–3.59; p < 0.0001; *I* ^2^ = 0.0%; Figure 3 D). These findings indicate a consistent association between heterozygous SERPINA1 genotypes and advanced liver disease outcomes.

## 4 Discussion

This systematic review and meta‐analysis synthesized data from 26 studies (32 cohorts), comprising 27,933 heterozygous participants, to define the hepatic phenotype associated with heterozygous SERPINA1 genotypes. Heterozygotes had modestly higher liver enzyme concentrations and increased odds of biochemical abnormalities, but the clearest signal was for clinically relevant liver disease: fibrosis, cirrhosis, and liver transplantation were each approximately twice as common in heterozygotes compared with MM controls. These findings support the view that heterozygous SERPINA1 genotypes are bona fide clinically relevant liver susceptibility states rather than benign carrier states.

The population implications are substantial because MZ and SZ genotypes are common. The MZ genotype occurs in approximately 1 in 25 individuals of European ancestry [34], and global modeling suggests that more than 100 million people may carry the MZ genotype [35]. Even a moderate increase in individual risk could therefore translate into a large burden of under‐recognized liver disease. In this context, the pooled results of our meta‐analysis support reframing common SERPINA1 heterozygosity as one of the most prevalent Mendelian risk factors for liver disease. Together, these observations have direct implications for how clinicians, laboratories, and policy makers should communicate genetic risk and design chronic liver disease pathways for this group.

A key finding was that excess liver risk was not explained by greater metabolic burden. BMI and steatosis odds were comparable between heterozygotes and controls, and the prevalence of obesity and steatosis was broadly consistent with contemporary general population estimates [36, 37, 38]. One plausible explanation is that, in a number of included studies, MM comparators were selected or matched to carriers on baseline characteristics, including factors related to metabolic risk, which would reduce observable differences in BMI. By contrast, although liver enzyme differences were small in absolute magnitude, the odds of advanced liver outcomes were substantially increased. This dissociation suggests that routine metabolic risk factors alone do not account for the observed hepatic phenotype, and that mild biochemical abnormalities may underestimate the underlying risk in some heterozygous carriers.

The discordance between comparable metabolic and biochemical profile and liver outcomes strongly supports a direct pathogenic contribution of the Z allele to hepatocellular injury, rather than simple confounding by metabolic syndrome. Independent analyses of population‐wide datasets have found that SERPINA1 Z allele (rs28929474) is associated with higher transaminase concentrations [34, 39], and higher rates of cirrhosis [40]. Our findings therefore fit a model in which common metabolic comorbidities may be important cofactors but cannot, on their own, account for the observed excess liver injury in Z allele carriers.

The magnitude of risk observed for advanced liver outcomes was comparable to established genetic and environmental modifiers of chronic liver disease. The approximately two‐fold increased odds of fibrosis, cirrhosis, and liver transplantation in heterozygous SERPINA1 carriers are within the range reported for variants such as PNPLA3 I148M (rs738409) and TM6SF2 E167K (rs58542926). The PNPLA3 I148M variant, for example, confers roughly 1.7–2.1‐fold higher odds of cirrhosis in heterozygous GC carriers compared with wild‐type CC in meta‐analyses of alcoholic [41] and mixed‐etiology cohorts [42]. The TM6SF2 E167K variant shows a broadly similar order of effect. In chronic hepatitis C, a meta‐analysis of four studies (*n* = 4325) reported an OR for cirrhosis of 2.05 (95% CI 1.39–3.02) in pooled homozygotes (KK) and heterozygotes (EK) versus non‐carriers (EE) [43]. In alcohol‐related liver disease, genome‐wide association data indicate that E167K carriage has been shown to increase the odds of alcohol‐related cirrhosis by approximately 1.6‐fold [44]. The magnitude of risk associated with heterozygous SERPINA1 genotypes in our meta‐analysis is similar to that of key environmental determinants. In population‐based cohorts, obesity roughly doubles the risk of cirrhosis‐related hospitalization or death, and heavy alcohol use alone increases cirrhosis risk by about three‐to‐four‐fold [45]. Importantly, our analyses for liver outcomes showed low statistical heterogeneity, despite variation in geography, genotype and recruitment strategy, which increases confidence that these associations are not artefacts of a few extreme cohorts.

The biological plausibility of this association is strong. The Z allele promotes misfolding, polymerization, and hepatocellular retention of A1AT, leading to ER stress [46, 47, 48, 49], inflammation, and cell death [50]. Although polymer burden is lower in MZ than ZZ disease, intrahepatic studies show that M and Z A1AT can co‐assemble into heteropolymers, and human iPSC‐hepatocyte models indicate that a single Z allele is sufficient to induce polymer accumulation [51] and cellular stress [47]. These data support a modifier model in which heterozygous SERPINA1 status lowers the threshold for fibrosis, especially when combined with metabolic dysfunction, harmful alcohol use, or other chronic inflammatory liver insults.

Clinically, these findings challenge the practice of labelling MZ and SZ genotypes as benign carrier states. In the United Kingdom, for example, several regional clinical resources explicitly describe those with the MZ genotype as “carriers” with lower but “sufficient” A1AT levels and “no significant increased risk” of lung or liver disease, recommending only generic lifestyle advice. The absolute risk of advanced liver disease remains modest, and most heterozygotes will not develop cirrhosis or require transplantation. However, heterozygous status should not be dismissed when liver enzymes are abnormal, steatosis is present, or additional risk factors coexist. Our findings support incorporating MZ and SZ status into chronic liver disease risk stratification and adopting a lower threshold for non‐invasive fibrosis assessment in carriers with obesity, type 2 diabetes, harmful alcohol use, or other chronic liver disease cofactors.

This study has several strengths. It provides the largest quantitative synthesis to date of liver outcomes in heterozygous SERPINA1 genotypes, incorporates both population‐based and clinically ascertained cohorts, and includes a predominantly contemporary evidence base. Genotype confirmation by targeted SERPINA1 genotyping or proteotyping was required for inclusion, reducing misclassification that may have affected older literature. Sensitivity analyses by genotype and age group revealed no significant difference from the pooled estimates in the main findings. The low heterogeneity observed for comparative analyses of fibrosis, cirrhosis, and liver transplantation strengthens confidence that the association with advanced liver outcomes is not driven by a small number of extreme studies.

Several limitations should also be considered. Most contributing data were cross‐sectional or retrospective, limiting inference about age‐specific incidence, fibrosis progression, and the effect of modifying exposures such as weight loss or alcohol reduction. Adjustment for key confounders, including alcohol intake, metabolic syndrome, viral hepatitis, and autoimmune liver disease, varied across studies, and residual confounding remains possible. In pooled proportion analyses, high *I* ^2^ values were interpreted conservatively, as *I* ^2^ was developed in the context of comparative meta‐analysis and can be high when pooling proportions, without necessarily indicating true inconsistency between studies [52]. Therefore, heterogeneity was considered alongside the direction and precision of estimates, subgroup analyses, and clinical characteristics of the included populations.

Future research should define how risk varies across the life course, identify modifying factors, and test targeted interventions. Longitudinal prospective studies of SERPINA1 heterozygotes assessing alcohol use, diet, metabolic parameters, and non‐invasive fibrosis markers will be essential to provide absolute and conditional risk estimates suitable for counselling. Large disease‐specific registries, such as national A1ATD registries and rare‐liver networks, can complement these efforts by providing deep phenotyping, longitudinal outcomes, and access to biosamples in clinically enriched populations. In parallel, unbiased population‐scale resources such as UK Biobank, All of Us and other linked electronic health‐record cohorts will remain crucial for testing gene–environment interactions, refining risk prediction algorithms and examining competing risks at scale. Mechanistic studies using iPSC‐derived hepatocytes, organoids, and in vivo models should dissect how heterozygous polymer load interacts with steatosis, insulin resistance, and immunological stimuli to drive fibrogenesis, potentially revealing therapeutic targets that extend beyond A1ATD. Pragmatic clinical trials could then evaluate whether metabolic interventions or emerging polymer‐targeted therapies in MZ carriers with early liver injury prevent progression more effectively than standard care. Finally, health economic modeling is needed to determine the cost‐effectiveness of incorporating SERPINA1 heterozygosity into routine chronic liver disease risk stratification, screening initiatives, and transplant allocation policies.

## Author Contributions

**Dimitra Georgantaki:** data curation, investigation, validation, supervision, writing – review and editing. **Riona T. T. Linn:** data curation, investigation, validation, writing – review and editing. **Amara Agbai:** data curation. **Mariam Molokhia:** supervision, project administration, conceptualization. **Adam M. Syanda:** conceptualization, methodology, software, investigation, validation, formal analysis, supervision, visualization, project administration, writing – original draft, writing – review and editing. **Richard Thompson:** supervision. **Manako Sakai:** data curation. **Hassan Tahir:** data curation. **S. Tamir Rashid:** conceptualization, supervision, funding acquisition, project administration, writing – review and editing.

## Funding

The authors have nothing to report.

## Conflicts of Interest

The authors declare no conflicts of interest.

## Supporting information


**Table S1:** Search terms used in the systematic review.
**Table S2:** Characteristics of included studies.
**Table S3:** PRISMA checklist.
**Figure S1:** Study selection and characteristics of included cohorts. (A) PRISMA flow diagram summarizing study identification, screening, eligibility assessment, and inclusion. (B) Cumulative number of eligible publications by year. (C) Sex distribution across SERPINA1 genotype groups, with random‐effects meta‐regression showing no significant association between female proportion and genotype. (D) Geographic distribution of included cohorts by country or region.
**Figure S2:** Study quality assessment using the Newcastle–Ottawa Scale. Non‐randomized studies were scored from 0 to 9 across the domains of selection, comparability, and outcome. Total scores were categorized as high (7–9), fair (4–6), or low (0–3) quality.
**Figure S3:** Leave‐one‐out sensitivity analysis. Panels show the impact of excluding individual studies on pooled estimates for (A) comorbidities associated with metabolic syndrome (obesity, type 2 diabetes, steatosis), (B) serum liver enzymes (ALT, AST, ALP), and (C) liver disease outcomes (fibrosis, cirrhosis, liver transplantation). Each point represents the pooled estimate recalculated after omitting the indicated study. The green band represents the 95% CI of the complete meta‐analysis. Labels indicate whether the leave‐one‐out pooled estimate differed from the complete‐set pooled estimate using a two‐sided z test. Exclusion of any single study does not materially change the summary estimates for any outcome, ns = *p* > 0.05, * = *p* < 0.05.
**Figure S4:** Sensitivity analysis of metabolic comorbidities and hepatic steatosis stratified by SERPINA1 genotype. Pooled estimates are presented by genotype subgroup for obesity prevalence (A), BMI mean difference versus MM controls (B), type 2 diabetes prevalence (C), steatosis prevalence (D), and steatosis odds ratio versus MM controls (E). Subgroup estimates are shown for MZ, SZ, and combined heterozygous groups where data were available. Stratification by MZ, SZ, and combined heterozygous groups did not materially alter the overall finding in Figure 1. The dashed line indicates the pooled estimate and the green shaded area the pooled 95% CI. BMI = body mass index; CI = confidence interval; HET = heterozygous; MD = mean difference; OR = odds ratio.
**Figure S5:** Sensitivity analysis of liver biochemistry stratified by SERPINA1 genotype. Genotype‐stratified random‐effects meta‐analyses are presented for ALT mean difference (A), elevated ALT odds ratio (B), AST mean difference (C), elevated AST odds ratio (D), ALP mean difference (E), and elevated ALP odds ratio (F). Stratification by MZ, SZ, and combined heterozygous groups supported the overall finding of modestly higher liver biochemistry abnormalities in heterozygotes compared with MM controls. The dashed line indicates the pooled estimate and the green shaded area the pooled 95% CI. ALT = alanine aminotransferase; ALP = alkaline phosphatase; AST = aspartate aminotransferase; CI = confidence interval; HET = heterozygous; MD = mean difference; OR = odds ratio.
**Figure S6:** Sensitivity analysis of fibrosis, cirrhosis, and liver transplantation stratified by SERPINA1 genotype. Genotype‐stratified random‐effects meta‐analyses are presented for fibrosis prevalence (A), fibrosis odds ratio (B), cirrhosis odds ratio (C), and liver transplantation odds ratio (D). Stratification by MZ, SZ, and combined heterozygous groups supported the overall finding of increased odds of fibrosis, cirrhosis, and liver transplantation in heterozygotes compared with MM controls. The dashed line indicates the pooled estimate and the green shaded area the corresponding 95% CI. CI = confidence interval; HET = heterozygous; OR = odds ratio.
**Figure S7:** Sensitivity analysis of liver outcomes stratified by age group. Age‐stratified random‐effects meta‐analyses are presented for outcomes from studies reporting both adult and pediatric cohorts: BMI mean difference (A), ALT mean difference (B), AST mean difference (C), and liver transplantation odds ratio (D). Studies reporting only adult cohorts were not included in this sensitivity analysis. Pediatric subgroup estimates were limited by small numbers of cohorts. The dashed line indicates the pooled estimate and the green shaded area the corresponding 95% CI. ALT = alanine aminotransferase; AST = aspartate aminotransferase; BMI = body mass index; CI = confidence interval; HET = heterozygous; MD = mean difference; OR = odds ratio.
**Figure S8:** Assessment of publication bias and small‐study effects. Funnel plots and Egger regression analyses shown for each meta‐analyzed outcome. Funnel plots display study‐level effect estimates against their standard errors, with the random‐effects pooled estimate shown as the central reference line and pseudo‐95% confidence limits around the pooled effect indicated by the shaded region. Egger regression plots show the standardized effect estimate against study precision (1/SE), with the fitted regression line shown in blue and the Egger test *p* value reported for each outcome. Analyses were performed on the appropriate model scale: logit‐transformed prevalence for prevalence outcomes, mean difference for continuous outcomes, and log odds ratio for binary comparative outcomes.

## Data Availability

All data used in this study are extracted from published or publicly available sources; the curated extraction dataset and Python analysis code will be made available by the corresponding author on reasonable request.
