# Supplementary material for: Meta‐Analysis: Redefining Liver Disease Risk in Heterozygous Alpha‐1 Antitrypsin Deficiency
Source: Aliment Pharmacol Ther. 2026 Jul 12;64(4):430–40. doi: 10.1111/apt.70814 (PMC13419271; doi:10.1111/apt.70814)
Supplement: Supplementary file 1 — Table S1: Search terms used in the systematic review. Table S2: Characteristics of included studies. Table S3: PRISMA checklist. Figure S1: Study selection and characteristics of included cohorts. (A) PRISMA flow diagram summarizing study identification, screening, eligibility assessment, and inclusion. (B) Cumulative number of eligible publications by year. (C) Sex distribution across SERPINA1 genotype groups, with random‐effects meta‐regression showing no significant association between female proportion and genotype. (D) Geographic distribution of included cohorts by country or region. Figure S2: Study quality assessment using the Newcastle–Ottawa Scale. Non‐randomized studies were scored from 0 to 9 across the domains of selection, comparability, and outcome. Total scores were categorized as high (7–9), fair (4–6), or low (0–3) quality. Figure S3: Leave‐one‐out sensitivity analysis. Panels show the impact of excluding individual studies on pooled estimates for (A) comorbidities associated with metabolic syndrome (obesity, type 2 diabetes, steatosis), (B) serum liver enzymes (ALT, AST, ALP), and (C) liver disease outcomes (fibrosis, cirrhosis, liver transplantation). Each point represents the pooled estimate recalculated after omitting the indicated study. The green band represents the 95% CI of the complete meta‐analysis. Labels indicate whether the leave‐one‐out pooled estimate differed from the complete‐set pooled estimate using a two‐sided z test. Exclusion of any single study does not materially change the summary estimates for any outcome, ns = p > 0.05, * = p < 0.05. Figure S4: Sensitivity analysis of metabolic comorbidities and hepatic steatosis stratified by SERPINA1 genotype. Pooled estimates are presented by genotype subgroup for obesity prevalence (A), BMI mean difference versus MM controls (B), type 2 diabetes prevalence (C), steatosis prevalence (D), and steatosis odds ratio versus MM controls (E). Subgroup estimates are shown for MZ, SZ, and combin [file APT-64-430-s001.zip › apt70814-sup-0001-Supinfo01/Supplementary Figure S2.pdf]

Supplementary Figure S2: Study quality assessment using the Newcastle–Ottawa Scale. Non-randomized studies were scored from 0 to 9 across the domains of selection, comparability, and outcome. Total scores were categorized as high (7–9), fair (4–6), or low (0–3) quality.

	Questions	Scoring
Selection	Is sample representative of A1ATD heterozygous cohort?<br>*(maximum 1 point)*	A: Fully representative **(1 point)**<br>B: Somewhat representative **(1 point)**<br>C: Not representative (0 points)<br>D: No description (0 points; unknown)
	Is control cohort drawn from the same community as the exposed cohort?<br>*(maximum 1 point)*	A: Drawn from the same community as the exposed cohort **(1 point)**<br>B: Drawn from a similar but not identical source (e.g. different clinic in the same healthcare system) (0 points)<br>B: Drawn from a different source (0 points)<br>C: No description (0 points; unknown)
	How was the A1ATD genotype ascertained?<br>*(maximum 1 point)*	A: Clinically diagnosed cases by genotype/phenotype **(1 point)**<br>B: Existing registry data (0 points)<br>C: Self-reported (0 points)<br>D: No description (0 points; unknown)
	Was the outcome of interest present at the start of the study?<br>*(maximum 1 point)*	A: Yes **(1 point)**<br>B: No (0 points)
Comparability	Were cohorts comparable on the basis of the design or analysis?<br>*(maximum 2 points)*	A: The study controls for age and sex **(1 point)**<br>B: Study controls for additional factors (e.g. age, sex) **(1 point)**<br>C: Cohorts are not comparable; no controls described (0 points)
Outcome	How was outcome of interest ascertained?<br>*(maximum 1 point)*	A: Structured clinical assessment **(1 point)**<br>B: Clinical record **(1 point)**<br>C: Self-report (0 points)<br>D: No description (0 points; unknown)
	Was follow-up long enough for outcomes to occur?<br>*(maximum 1 point)*	A: Yes **(1 point)**<br>B: No or not described (0 points)
	Was follow-up cohort adequate?<br>*(maximum 1 point)*	A: Complete follow up for all **(1 point)**<br>B: ≤20% follow up dropout **(1 point)**<br>C: ≥20% follow up dropout (0 points)<br>D: No statement (0 points; unknown)<br>E: No follow-up (0 points)

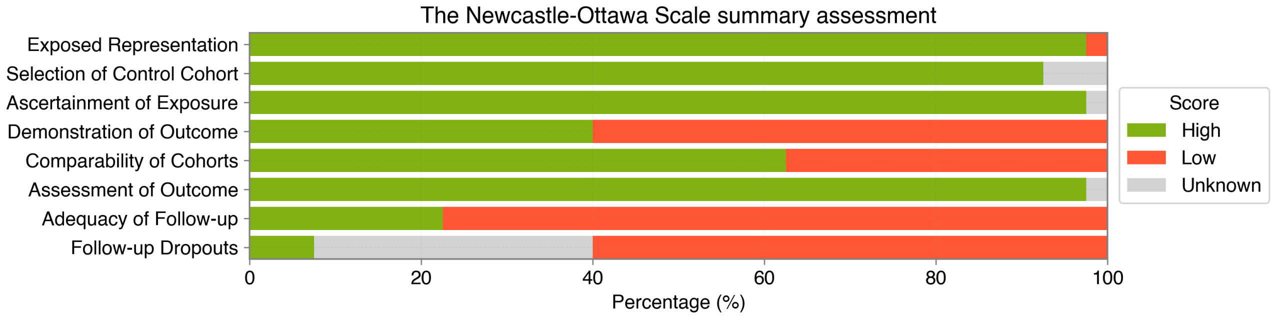

Score: 1 = +; 0 = -; Unknown (0) = ?

Cohort	Selection				Comparability	Outcome				
	Exposed Representation	Selection of Control Cohort	Ascertainment of Exposure	Demonstration of Outcome	Comparability of Cohorts	Assessment of Outcome	Adequacy of Follow-up	Follow-up Dropouts	Score	Study quality
Aiello (2022a)	+	+	+	-	-	+	-	-	4	Fair
Barjaktarevic (2025)	+	+	+	+	+	+	+	?	7	High
Bernspang (2009)	+	+	+	+	+	+	+	?	7	High
Campbell (2007a)	+	+	+	-	-	+	-	-	4	Fair
Campbell (2007b)	+	+	+	-	-	+	-	-	4	Fair
Carlson (1985)	+	?	+	+	+	+	+	?	6	Fair
Chen (2022)	+	+	+	+	-	+	+	+	7	High
Choate (2019)	+	+	+	+	+	?	-	-	5	Fair
Escribano (2016)	+	+	+	-	+	+	-	-	5	Fair
Franciosi (2020a)	+	+	+	+	+	+	-	-	6	Fair
Franciosi (2020b)	+	+	+	+	+	+	+	?	7	High
Fromme (2024b)	+	+	+	-	+	+	-	-	5	Fair
Fromme (2024c)	+	+	+	-	+	+	-	-	5	Fair
Fromme (2024d)	+	+	+	-	+	+	-	-	5	Fair
Fromme (2024a)	+	+	+	+	-	+	+	?	6	Fair
Fromme (2022b)	+	+	+	+	+	+	-	-	6	Fair
Fromme (2022c)	+	?	+	-	+	+	-	-	4	Fair
Fromme (2022a)	+	+	+	+	+	+	-	-	6	Fair
Goltz (2014)	+	+	+	-	+	+	-	-	5	Fair
Gupta (2020)	+	+	+	-	+	+	-	-	5	Fair
Hashemi (2005)	+	?	+	-	+	+	-	-	4	Fair
Mandorfer (2018)	+	+	+	-	+	+	-	-	5	Fair
Molloy (2014)	+	+	+	-	+	+	-	-	5	Fair
Murali (2023a)	+	+	+	-	+	+	-	-	5	Fair
Pons (2021)	+	+	?	+	+	+	-	-	5	Fair
Riis (2022)	+	+	+	-	-	+	+	+	6	Fair
Schneider (2020a)	+	+	+	+	+	+	-	?	6	Fair
Schneider (2020b)	+	+	+	+	+	+	-	?	6	Fair
Schneider (2020c)	+	+	+	+	+	+	-	?	6	Fair
Schuler (2023a)	+	+	+	-	-	+	-	?	4	Fair
Schuler (2023b)	+	+	+	-	-	+	-	?	4	Fair
Schuler (2023c)	+	+	+	-	-	+	-	?	4	Fair
Serban (2022)	-	+	+	-	+	+	-	?	4	Fair
Strnad (2019a)	+	+	+	-	-	+	-	-	4	Fair
Strnad (2019b)	+	+	+	-	-	+	-	-	4	Fair
Strnad (2019c)	+	+	+	-	-	+	-	-	4	Fair
Strnad (2019d)	+	+	+	-	-	+	-	-	4	Fair
Strnad (2019e)	+	+	+	-	-	+	-	-	4	Fair
Tanash (2015)	+	+	+	+	-	+	+	+	7	High
Thun (2012)	+	+	+	+	+	+	+	?	7	High
