# Supplementary material for: Meta‐Analysis: Redefining Liver Disease Risk in Heterozygous Alpha‐1 Antitrypsin Deficiency
Source: Aliment Pharmacol Ther. 2026 Jul 12;64(4):430–40. doi: 10.1111/apt.70814 (PMC13419271; doi:10.1111/apt.70814)
Supplement: Supplementary file 1 — Table S1: Search terms used in the systematic review. Table S2: Characteristics of included studies. Table S3: PRISMA checklist. Figure S1: Study selection and characteristics of included cohorts. (A) PRISMA flow diagram summarizing study identification, screening, eligibility assessment, and inclusion. (B) Cumulative number of eligible publications by year. (C) Sex distribution across SERPINA1 genotype groups, with random‐effects meta‐regression showing no significant association between female proportion and genotype. (D) Geographic distribution of included cohorts by country or region. Figure S2: Study quality assessment using the Newcastle–Ottawa Scale. Non‐randomized studies were scored from 0 to 9 across the domains of selection, comparability, and outcome. Total scores were categorized as high (7–9), fair (4–6), or low (0–3) quality. Figure S3: Leave‐one‐out sensitivity analysis. Panels show the impact of excluding individual studies on pooled estimates for (A) comorbidities associated with metabolic syndrome (obesity, type 2 diabetes, steatosis), (B) serum liver enzymes (ALT, AST, ALP), and (C) liver disease outcomes (fibrosis, cirrhosis, liver transplantation). Each point represents the pooled estimate recalculated after omitting the indicated study. The green band represents the 95% CI of the complete meta‐analysis. Labels indicate whether the leave‐one‐out pooled estimate differed from the complete‐set pooled estimate using a two‐sided z test. Exclusion of any single study does not materially change the summary estimates for any outcome, ns = p > 0.05, * = p < 0.05. Figure S4: Sensitivity analysis of metabolic comorbidities and hepatic steatosis stratified by SERPINA1 genotype. Pooled estimates are presented by genotype subgroup for obesity prevalence (A), BMI mean difference versus MM controls (B), type 2 diabetes prevalence (C), steatosis prevalence (D), and steatosis odds ratio versus MM controls (E). Subgroup estimates are shown for MZ, SZ, and combin [file APT-64-430-s001.zip › apt70814-sup-0001-Supinfo01/Supplementary Table S1.pdf]

Search strategy.

OVID databases

- MEDLINE (Ovid)
- EMBASE Classic and EMBASE (Ovid)
- Global Health (Ovid)
- Journals@Ovid (Ovid)

Step	Search term
1	(a-atd or a1-atd or a1at or a1atd or aatd or anti-tryps$ or antitryps$ or one-anti-tryps$ or one-antitryps$ or anti-proteinas$ or antiproteinas$ or alpha-1-anti-proteinas$ or alpha-1-anti-tryps$ or alfa-1-anti-tryps$ or alfa-1-antitryps$ or alpha-1-antiproteinas$ or alpha-1-antitryps$ or alpha-1-at or alpha-1-atd or alpha-one-anti-tryps$ or alpha-one-antitryps$ or alpha1-anti-proteinas$ or alpha1-anti-tryps$ or alpha1-antiproteinas$ or alpha1-antitryps$ or alpha1-at or proteinase inhibito$ or alpha1-proteinase inhibito$ or alpha-1-proteinase inhibito$ or 1-proteinase inhibito$ or one-proteinase inhibito$ or alpha1-atd or pimz or mz or SERPINA1).ti,ab.
2	1 and (defic$ or lack$).ti,ab.
3	2 not (exp animals/ not humans.sh.)
4	3 not (mouse or murine or mice or "in vitro" or model).ti,ab.
5	4 and (OLT or transplant$ or morb$ or comorb$ or morbidi$ or decompensat$ or fail$ or diseas$ or disord$ or patho$ or abnorm$ or indication$ or hepatit$ or hepato$ or carcinom$ or cance$ or risk$ or prevalen$ or inciden$ or predict$ or associate$ or liver or cirr$ or fibro$).ti,ab.
6	5 not review.pt.
7	remove duplicates from 6

Cochrane Library: Reviews, Protocols, Clinical Trials

Step	Search term
#1	(a1at or a1atd or aatd or anti-tryps* or antitryps* or one-anti-tryps* or one-antitryps* or anti-proteinase or antiproteinase or pimz or " mz " or SERPINA1):ti,ab,kw
#2	(defic* OR lack*):ti,ab,kw
#3	#1 and #2
#4	(OLT or transplant* or morb* or comorb* or morbidi* or decompensat* or fail* or diseas* or disord* or patho* or abnorm* or indication* or hepatit* or hepato* or carcinom* or cance* or risk* or prevalen* or inciden* or predict* or associated or liver or cirr* or fibro*):ti,ab,kw
#5	#3 and #4

EU Clinical Trials Register

Search term

anti-trypsin deficiency OR antitrypsin deficiency OR pimz OR SERPINA1

NHS EED/DARE/HTA

**Search term (Title)**
anti-trypsin OR antitrypsin OR SERPINA1

EBSCO database - CINAHL (EBSCO)

**Step**	**Search term**	**Search Options**
S1	TI (a-atd or a1-atd or a1at or a1atd or aatd or anti-tryps* or antitryps* or one-anti-tryps* or one-antitryps* or anti-proteinase or antiproteinase or alpha-1-anti-proteinas* or alpha-1-anti-tryps* or alfa-1-anti-tryps* or alfa-1-antitryps* or alpha-1-antiproteinas* or alpha-1-antitrypsin or alpha-1-at or alpha-1-atd or alpha-one-anti-trypsin or alpha-one-antitrypsin or alpha1-anti-proteinase or alpha1-anti-trypsin or alpha1-antiproteinase or alpha1-antitryps* or alpha1-at or proteinase inhibito* or alpha1-proteinase inhibito* or alpha-1-proteinase inhibito* or 1-proteinase inhibito* or one-proteinase inhibito* or alpha1-atd or pimz or pimz or SERPINA1)	**Expanders** - Apply related words; Apply equivalent subjects<br>**Search modes** - Boolean/Phrase
S2	AB (a-atd or a1-atd or a1at or a1atd or aatd or anti-tryps* or antitryps* or one-anti-tryps* or one-antitryps* or anti-proteinase or antiproteinase or alpha-1-anti-proteinas* or alpha-1-anti-tryps* or alfa-1-anti-tryps* or alfa-1-antitryps* or alpha-1-antiproteinas* or alpha-1-antitrypsin or alpha-1-at or alpha-1-atd or alpha-one-anti-trypsin or alpha-one-antitrypsin or alpha1-anti-proteinase or alpha1-anti-trypsin or alpha1-antiproteinase or alpha1-antitryps* or alpha1-at or proteinase inhibito* or alpha1-proteinase inhibito* or alpha-1-proteinase inhibito* or 1-proteinase inhibito* or one-proteinase inhibito* or alpha1-atd or pimz or mz or SERPINA1)	**Expanders** - Apply related words; Apply equivalent subjects<br>**Search modes** - Boolean/Phrase
S3	S1 or S2	**Expanders** - Apply equivalent subjects<br>**Search modes** - Boolean/Phrase
S4	TI (defic* or lack*)	**Expanders** - Apply related words; Apply equivalent subjects<br>**Search modes** - Boolean/Phrase
S5	AB (defic* or lack*)	**Expanders** - Apply related words; Apply equivalent subjects<br>**Search modes** - Boolean/Phrase
S6	S4 or S5	**Expanders** - Apply equivalent subjects<br>**Search modes** - Boolean/Phrase

S7	S3 and S6	**Expanders** - Apply equivalent subjects<br>**Search modes** - Boolean/Phrase
S8	TI (OLT or transplant* or morb* or comorb* or morbidi* or decompensat* or fail* or diseas* or disord* or patho* or abnorm* or indication* or hepatit* or hepato* or carcinom* or cance* or risk* or prevalen* or inciden* or predict* or associated or liver or cirr* or fibro*)	**Expanders** - Apply related words; Apply equivalent subjects<br>**Search modes** - Boolean/Phrase
S9	AB (OLT or transplant* or morb* or comorb* or morbidi* or decompensat* or fail* or diseas* or disord* or patho* or abnorm* or indication* or hepatit* or hepato* or carcinom* or cance* or risk* or prevalen* or inciden* or predict* or associated or liver or cirr* or fibro*)	**Expanders** - Apply related words; Apply equivalent subjects<br>**Search modes** - Boolean/Phrase
S10	S8 or S9	**Expanders** - Apply equivalent subjects<br>**Search modes** - Boolean/Phrase
S11	S7 and S10	**Expanders** - Apply equivalent subjects<br>**Search modes** - Boolean/Phrase
S12	S11 NOT (((MH "Animals+") OR (MH "Animal Studies") OR (TI "animal model*")) NOT (MH "human"))	**Expanders** - Apply equivalent subjects<br>**Search modes** - Boolean/Phrase
S13	S12 NOT (mouse or murine or mice or "in vitro" or model)	**Expanders** - Apply related words; Apply equivalent subjects<br>**Search modes** - Boolean/Phrase

PubMed Central

Step	Search term
#1	a-atd[Abstract] OR a1-atd[Abstract] OR a1at[Abstract] OR a1atd[Abstract] OR aatd[Abstract] OR anti-tryps*[Abstract] OR antitryps*[Abstract] OR one-anti-tryps*[Abstract] OR one-antitryps*[Abstract] OR anti-proteinase[Abstract] OR antiproteinase[Abstract] OR alpha-1-anti-proteinas*[Abstract] OR alpha-1-anti-tryps*[Abstract] OR alfa-1-anti-tryps*[Abstract] OR alfa-1-antitryps*[Abstract] OR alpha-1-antiproteinas*[Abstract] OR alpha-1-antitrypsin[Abstract] OR alpha-1-at[Abstract] OR alpha-1-atd[Abstract] OR alpha-one-anti-trypsin[Abstract] OR alpha-one-antitrypsin[Abstract] OR alpha1-anti-proteinase[Abstract] OR alpha1-anti-trypsin[Abstract] OR alpha1-antiproteinase[Abstract] OR alpha1-antitryps*[Abstract] OR alpha1-at[Abstract] OR proteinase inhibito*[Abstract] OR alpha1-proteinase inhibito*[Abstract] OR alpha-1-proteinase inhibito*[Abstract] OR 1-proteinase inhibito*[Abstract] OR proteinase inhibito*[Abstract] OR alpha1-atd[Abstract] OR pimz[Abstract] OR mz[Abstract] OR SERPINA1[Abstract]
#2	a-atd[Title] OR a1-atd[Title] OR a1at[Title] OR a1atd[Title] OR aatd[Title] OR anti-tryps*[Title] OR antitryps*[Title] OR one-anti-tryps*[Title] OR one-antitryps*[Title] OR anti-proteinase[Title] OR antiproteinase[Title] OR alpha-1-anti-proteinas*[Title] OR alpha-1-anti-tryps*[Title] OR alfa-1-anti-tryps*[Title] OR alfa-1-antitryps*[Title] OR alpha-1-antiproteinas*[Title] OR alpha-1-antitrypsin[Title] OR alpha-1-at[Title] OR alpha-1-atd[Title] OR alpha-one-anti-trypsin[Title] OR alpha-one-antitrypsin[Title] OR alpha1-anti-proteinase[Title] OR alpha1-anti-trypsin[Title] OR alpha1-antiproteinase[Title] OR alpha1-antitryps*[Title] OR alpha1-at[Title] OR proteinase inhibito*[Title] OR alpha1-proteinase inhibito*[Title] OR alpha-1-proteinase inhibito*[Title] OR

	1-proteinase inhibito*[Title] OR one-proteinase inhibito*[Title] OR alpha1-atd[Title] OR pimz[Title] OR mz[Title] OR SERPINA1[Title]
#3	#1 or #2
#4	#3 AND ((defic*[Abstract] OR lack*[Abstract]) OR (defic*[Title] OR lack*[Title]))
#5	OLT[Abstract] OR transplant*[Abstract] OR morb*[Abstract] OR comorb*[Abstract] OR morbidi*[Abstract] OR decompensat*[Abstract] OR fail*[Abstract] OR disease[Abstract] OR diseases[Abstract] OR disord*[Abstract] OR pathol*[Abstract] OR abnorm*[Abstract] OR indication*[Abstract] OR hepatit*[Abstract] OR hepatoc*[Abstract] OR carcinom*[Abstract] OR cancer*[Abstract] OR risk*[Abstract] OR prevalen*[Abstract] OR inciden*[Abstract] OR predict*[Abstract] OR associated[Abstract] OR liver[Abstract] OR cirr*[Abstract] OR fibro*[Abstract]
#6	OLT[Title] OR transplant*[Title] OR morb*[Title] OR comorb*[Title] OR morbidi*[Title] OR decompensat*[Title] OR fail*[Title] OR disease[Title] OR diseases[Title] OR disord*[Title] OR pathol*[Title] OR abnorm*[Title] OR indication*[Title] OR hepatit*[Title] OR hepatoc*[Title] OR carcinom*[Title] OR cancer*[Title] OR risk*[Title] OR prevalen*[Title] OR inciden*[Title] OR predict*[Title] OR associated[Title] OR liver[Title] OR cirr*[Title] OR fibro*[Title]
#7	#5 OR #6
#8	#4 AND #7
#9	#8 NOT (animals [mh] NOT humans [mh])
#10	mouse[Abstract] OR mice[Abstract] OR murine[Abstract] OR “in vitro”[Abstract] OR model[Abstract]
#11	mouse[Title] OR mice[Title] OR murine[Title] OR “in vitro”[Title] OR model[Title]
#12	#10 OR #11
#13	#9 NOT #12
#14	#13 NOT "is retracted"[filter]

PubMed

Step	Search term
#1	a-atd[Title/Abstract] OR a1-atd[Title/Abstract] OR a1at[Title/Abstract] OR a1atd[Title/Abstract] OR aatd[Title/Abstract] OR anti-tryps*[Title/Abstract] OR antitryps*[Title/Abstract] OR one-anti-tryps*[Title/Abstract] OR one-antitryps*[Title/Abstract] OR anti-proteinase[Title/Abstract] OR antiproteinase[Title/Abstract] OR alpha-1-anti-proteinas*[Title/Abstract] OR alpha-1-anti-tryps*[Title/Abstract] OR alfa-1-anti-tryps*[Title/Abstract] OR alfa-1-antitryps*[Title/Abstract] OR alpha-1-antiproteinas*[Title/Abstract] OR alpha-1-antitrypsin[Title/Abstract] OR alpha-1-at[Title/Abstract] OR alpha-1-atd[Title/Abstract] OR alpha-one-anti-trypsin[Title/Abstract] OR alpha-one-antitrypsin[Title/Abstract] OR alpha1-anti-proteinase[Title/Abstract] OR alpha1-anti-trypsin[Title/Abstract] OR alpha1-antiproteinase[Title/Abstract] OR alpha1-antitryps*[Title/Abstract] OR alpha1-at[Title/Abstract] OR proteinase inhibito*[Title/Abstract] OR alpha1-proteinase inhibito*[Title/Abstract] OR alpha-1-proteinase inhibito*[Title/Abstract] OR 1-proteinase inhibito*[Title/Abstract] OR proteinase inhibito*[Title/Abstract] OR alpha1-atd[Title/Abstract] OR pimz[Title/Abstract] OR mz[Title/Abstract] OR SERPINA1[Title/Abstract]

#2	#1 AND (defic*[Title/Abstract] OR lack*[Title/Abstract])
#3	#2 AND (OLT[Title/Abstract] OR transplant*[Title/Abstract] OR morb*[Title/Abstract] OR comorb*[Title/Abstract] OR morbidi*[Title/Abstract] OR decompensat*[Title/Abstract] OR fail*[Title/Abstract] OR disease[Title/Abstract] OR diseases[Title/Abstract] OR disord*[Title/Abstract] OR pathol*[Title/Abstract] OR abnorm*[Title/Abstract] OR indication*[Title/Abstract] OR hepatit*[Title/Abstract] OR hepatoc*[Title/Abstract] OR carcinom*[Title/Abstract] OR cancer*[Title/Abstract] OR risk*[Title/Abstract] OR prevalen*[Title/Abstract] OR inciden*[Title/Abstract] OR predict*[Title/Abstract] OR associated[Title/Abstract]] OR liver[Title/Abstract] OR cirr*[Title/Abstract] OR fibro*[Title/Abstract])
#4	#3 NOT (animals [mh] NOT humans [mh])
#5	#4 NOT (mouse[Title/Abstract] OR mice[Title/Abstract] OR murine[Title/Abstract] OR "in vitro"[Title/Abstract] OR model[Title/Abstract])
#6	#5 NOT (comment[pt] OR review[pt] OR book[pt] OR booksdocs[pt])

Clinical Trials.gov

**Search term**
antitrypsin OR anti-trypsin OR SERPINA1

ISRCNT Registry

**Search term**
antitrypsin OR anti-trypsin AND deficiency OR SERPINA1

WHO ICTRP

**Search term**
(antitrypsin deficiency) OR (anti-trypsin deficiency) OR SERPINA1

MedRxiv

**Search term**
antitrypsin deficiency OR SERPINA1

Web of Science (Clarivate)

Step	Search term
#1	TI=(a-atd or a1-atd or a1at or a1atd or aatd or anti-tryps* or antitryps* or one-anti-tryps* or one-antitryps* or anti-proteinase or antiproteinase or alpha-1-anti-proteinas* or alpha-1-anti-tryps* or alfa-1-anti-tryps* or alfa-1-antitryps* or alpha-1-antiproteinas* or alpha-1-antitrypsin or alpha-1-at or alpha-1-atd or alpha-one-anti-trypsin or alpha-one-antitrypsin or alpha1-anti-proteinase or alpha1-anti-trypsin or alpha1-antiproteinase or alpha1-antitryps* or alpha1-at or proteinase inhibito* or alpha1-proteinase inhibito* or alpha-1-proteinase inhibito* or 1-proteinase inhibito* or one-proteinase inhibito* or alpha1-atd or pimz or mz or SERPINA1)
#2	AB=(a-atd or a1-atd or a1at or a1atd or aatd or anti-tryps* or antitryps* or one-anti-tryps* or one-antitryps* or anti-proteinase or antiproteinase or alpha-1-anti-proteinas* or alpha-1-anti-tryps* or alfa-1-anti-tryps* or alfa-1-antitryps* or alpha-1-antiproteinas* or alpha-1-antitrypsin or alpha-1-at or alpha-1-atd or alpha-one-anti-trypsin or alpha-one-antitrypsin or alpha1-anti-proteinase or alpha1-anti-trypsin or alpha1-antiproteinase or alpha1-antitryps* or alpha1-at or proteinase inhibito* or alpha1-proteinase inhibito* or alpha-1-proteinase inhibito* or 1-proteinase inhibito* or one-proteinase inhibito* or alpha1-atd or pimz or mz or SERPINA1)
#3	#1 OR #2
#4	TI=(defic* or lack*)
#5	AB=(defic* or lack*)
#6	#4 OR #5
#7	#3 AND #6
#8	TI=(OLT or transplant* or morb* or comorb* or morbidi* or decompensat* or fail* or diseas* or disord* or patho* or abnorm* or indication* or hepatit* or hepato* or carcinom* or cance* or risk* or prevalen* or inciden* or predict* or associated or liver or cirr* or fibro*)
#9	AB=(OLT or transplant* or morb* or comorb* or morbidi* or decompensat* or fail* or diseas* or disord* or patho* or abnorm* or indication* or hepatit* or hepato* or carcinom* or cance* or risk* or prevalen* or inciden* or predict* or associated or liver or cirr* or fibro*)
#10	#8 OR #9
#11	#7 AND #10
#12	TI=(mouse or murine or mice or "in vitro" or model)
#13	AB=(mouse or murine or mice or "in vitro" or model)
#14	#12 OR #13
#15	#11 NOT #14
