# Supplementary material for: Meta‐Analysis: Redefining Liver Disease Risk in Heterozygous Alpha‐1 Antitrypsin Deficiency
Source: Aliment Pharmacol Ther. 2026 Jul 12;64(4):430–40. doi: 10.1111/apt.70814 (PMC13419271; doi:10.1111/apt.70814)
Supplement: Supplementary file 1 — Table S1: Search terms used in the systematic review. Table S2: Characteristics of included studies. Table S3: PRISMA checklist. Figure S1: Study selection and characteristics of included cohorts. (A) PRISMA flow diagram summarizing study identification, screening, eligibility assessment, and inclusion. (B) Cumulative number of eligible publications by year. (C) Sex distribution across SERPINA1 genotype groups, with random‐effects meta‐regression showing no significant association between female proportion and genotype. (D) Geographic distribution of included cohorts by country or region. Figure S2: Study quality assessment using the Newcastle–Ottawa Scale. Non‐randomized studies were scored from 0 to 9 across the domains of selection, comparability, and outcome. Total scores were categorized as high (7–9), fair (4–6), or low (0–3) quality. Figure S3: Leave‐one‐out sensitivity analysis. Panels show the impact of excluding individual studies on pooled estimates for (A) comorbidities associated with metabolic syndrome (obesity, type 2 diabetes, steatosis), (B) serum liver enzymes (ALT, AST, ALP), and (C) liver disease outcomes (fibrosis, cirrhosis, liver transplantation). Each point represents the pooled estimate recalculated after omitting the indicated study. The green band represents the 95% CI of the complete meta‐analysis. Labels indicate whether the leave‐one‐out pooled estimate differed from the complete‐set pooled estimate using a two‐sided z test. Exclusion of any single study does not materially change the summary estimates for any outcome, ns = p > 0.05, * = p < 0.05. Figure S4: Sensitivity analysis of metabolic comorbidities and hepatic steatosis stratified by SERPINA1 genotype. Pooled estimates are presented by genotype subgroup for obesity prevalence (A), BMI mean difference versus MM controls (B), type 2 diabetes prevalence (C), steatosis prevalence (D), and steatosis odds ratio versus MM controls (E). Subgroup estimates are shown for MZ, SZ, and combin [file APT-64-430-s001.zip › apt70814-sup-0001-Supinfo01/Supplementary Table S2.pdf]

First Author	Year	Publication title	DOI	PMID	Study Design	Country	Cohort ID	Cohort description	Genotype	Cohort size (n)	Comp. size (n)	Prevalence statistics	Odds ratio and standardised mean difference statistics
Aiello	2022	Distribution of the Clinical Manifestations of Alpha-1 Antitrypsin Deficiency in Respiratory Outpatients from an Area of Northern Italy.	10.1159/000525549	35793662	Cohort Study Retrospective	IT	-	MZ patients from University Hospital of Parma	MZ	31	45	Steatosis: 6.40%	-
Barjaktarevic	2025	Longitudinal Outcomes in Pi*MZ Alpha-1 Antitrypsin–Deficient Individuals with Tobacco Smoking History from the SPIROMICS Cohort	10.1513/AnnalsATS.202411-1209OC	40068143	Cohort Study Prospective	US	-	MZ individuals with ≥20 pack-years smoking history from the SPIROMICS cohort	MZ	79	1856		**BMI MD**: -0.57 [95% CI: -0.97, -0.18]
Bernspang	2009	The liver in 30-year-old individuals with Alpha-1 Antitrypsin deficiency.	10.3109/00365520903296669	19891586	Cohort Study Prospective	SE	-	SZ individuals identified from Swedish Neonatal Screening	SZ	40	84	-	**BMI MD:** 1.75 [95% CI: -0.60, 4.10]; **ALT MD:** 6.90 [95% CI: 5.79, 8.01]; **Elevated ALT OR:** 1.76 [95% CI: 0.44, 6.93]; **AST MD:** 8.85 [95% CI: 8.24, 9.46]; **Elevated AST OR:** 2.16 [95% CI: 0.29, 15.90]
Campbell	2007	High prevalence of Alpha-1 Antitrypsin heterozygosity in children with chronic liver disease	10.1097/01.mpg.0000243434.54958.21	17204961	Cohort Study Retrospective	US	a	Heterozygous A1ATD patients with no biliary atresia from Cincinnati Children’s Hospital considered for liver transplants	Mixed het.	21	96	-	**Liver Transplant OR:** 1.50 [95% CI: 0.46, 4.87]
							b	Heterozygous A1ATD patients with biliary atresia from Cincinnati Children’s Hospital considered for liver transplants	Mixed het.	10	45	-	**Liver Transplant OR:** 8.72 [95% CI: 0.48, 159.67]
Carlson	1985	Chronic Cryptogenic Liver Disease and Malignant Hepatoma in Intermediate Alpha-1 Antitrypsin Deficiency Identified by a Pi Z-Specific Monoclonal Antibody	10.3109/00365528509088831	2996119	Cohort Study Prospective	SE	a	Heterozygous A1ATD patients from Malmö General Hospital undergoing evaluation of liver function and who received liver biopsies	Mixed het.	42	59	-	**Steatosis OR:**0.55 [95% CI: 0.25, 1.23]; **Cirrhosis OR:**1.36 [95% CI: 0.60, 3.05]
							b	Heterozygous A1ATD patients with liver disease from Malmö General Hospital	Mixed het.	64	128	-	**Cirrhosis OR:**1.58 [95% CI: 0.80, 3.13]
Chen	2022	Hepatic decompensation is accelerated in patients with cirrhosis and Alpha-1 Antitrypsin Pi*MZ genotype.	10.1016/j.jhepr.2022.100483	35571533	Cohort Study Retrospective	US	-	MZ individuals from the Michigan Genomics Initiative	MZ	49	474	-	**BMI MD:** 0.28 [95% CI: -0.64, 1.24]; **ALT MD:** -5.50 [95% CI: -8.16, -2.84]; **AST MD:** 2.38 [95% CI: -0.35, 5.10]; **ALP MD:** 2.12 [95% CI: -4.44, 8.69]
Choate	2019	Comparing Patients with ZZ Versus SZ Alpha-1 Antitrypsin Deficiency: Findings	10.15326/jcopdf.6.1.2018.0134	30775422	Cohort Study Retrospective	US	-	SZ members of AlphaNet	SZ	504	3,031	Diabetes: 17.00%	-
Escribano	2016	Accelerated telomere attrition in children and teenagers with Alpha-1 Antitrypsin deficiency.	10.1183/13993003.00176-2016	27390278	Cohort Study Retrospective	ES	-	Heterozygous A1ATD patients from Hospital Clinico Universitario	Mixed Het.	34	18	-	**BMI MD**: 0.50 [95% CI: -0.87, 1.87]; **ALT MD:** 1.25 [95% CI: -2.89, 5.39]; **AST MD:** 1.50 [95% CI: -1.36, 4.36]

First Author	Year	Publication title	DOI	PMID	Study Design	Country	Cohort ID	Cohort description	Genotype	Cohort size (n)	Comp. size (n)	Prevalence statistics	Odds ratio and standardised mean difference statistics
								Valencia and Hospital Universitari Doctor Peset					
Franciosi	2020	Clarifying the Risk of Lung Disease in SZ Alpha-1 Antitrypsin Deficiency.	10.1164/rccm.202002-0262OC	32197047	Cohort Study Prospective	IE	a	MZ Family Members of SZ Individuals identified through Irish National AATD Targeted Detection Programme	MZ	28	19	-	**BMI MD:** 1.93 [95% CI: -0.60, 4.46]; **ALT MD:** -3.06 [95% CI: -5.35, -0.78]; **ALP MD:** 8.38 [95% CI: 2.69, 14.06]
							b	SZ individuals identified through Irish National AATD Targeted Detection Programme	SZ	70	19	-	**BMI MD:** 0.97 [95% CI: -0.84, 2.78]; **ALT MD:** 2.44 [95% CI: 0.26, 4.62]; **ALP MD:** 6.06 [95% CI: 0.85, 11.28]
Fromme	2022	Hepatobiliary phenotypes of adults with Alpha-1 Antitrypsin deficiency.	10.1136/gutjnl-2020-323729	33632708	Cohort Study Retrospective	GB	a	MZ individuals from UK Biobank	MZ	17,006	422,506	Obesity: 30.00%	**Cirrhosis OR:**1.66 [95% CI: 1.25, 2.21]
						GB	b	SZ individuals from UK Biobank	SZ	864	422,506	Obesity: 29.00% Diabetes: 3.59%	**ALT MD:** 1.30 [95% CI: 0.63, 1.97]; **Elevated ALT OR:** 1.41 [95% CI: 1.11, 1.79]; **AST MD:** 1.01 [95% CI: 0.36, 1.66]; **Elevated AST OR:** 1.22 [95% CI: 0.91, 1.64]; **ALP MD:** -2.23 [95% CI: -4.52, 0.06]; **Elevated ALP OR:** 1.49 [95% CI: 1.24, 1.79]; **Cirrhosis OR:** 2.62 [95% CI: 0.98, 7.02]
						EU	c	SZ individuals from Global Alpha-1 Liver initiative	SZ	239	279	Obesity: 23.10% Diabetes: 7.00% Steatosis: 39.30% Fibrosis: 11.01% Cirrhosis: 3.21%	**BMI MD:** 1.00 [95% CI: 0.13, 1.87]; **Steatosis OR:** 1.50 [95% CI: 1.05, 2.17]; **Elevated ALT OR:** 1.41 [95% CI: 0.85, 2.35]; **Elevated AST OR:** 2.41 [95% CI: 1.23, 4.71]; **Elevated ALP OR:** 7.81 [95% CI: 3.22, 18.91]; **Fibrosis OR:**1.96 [95% CI: 1.01, 3.84]; **Cirrhosis OR:**2.78 [95% CI: 0.71, 10.86]
Fromme	2024	Association of circulating Z-polymer with adverse clinical outcomes and liver fibrosis in adults with Alpha-1 Antitrypsin deficiency.	10.1002/ueg2.12629	39024029	Cohort Study Prospective	EU	a	MZ individuals from European Alpha-1 Liver Cohort	MZ	405	312	Diabetes: 3.46%	-
Fromme	2024	Alcohol consumption and liver phenotype of individuals with alpha-1 antitrypsin deficiency.	10.1111/liv.16044	39031304	Cohort Study Retrospective	GB	b	MZ individuals with no-alcohol/low alcohol intake from UK Biobank	MZ	14,304	351,967	-	**BMI MD:** -0.15 [95% CI: -0.37, 0.07]; **ALT MD:** 2.20 [95% CI: 2.15, 2.25]; **Elevated ALT OR:** 1.17 [95% CI: 1.10, 1.25]; **AST MD:** 0.61 [95% CI: 0.58, 0.64]; **Elevated AST OR:** 1.15 [95% CI: 1.06, 1.24]; **ALP MD:** 2.97 [95% CI: 2.83, 3.11]; **Elevated ALP OR:** 1.27 [95% CI: 1.21, 1.33]
						GB	c	MZ individuals with medium alcohol intake from UK Biobank	MZ	153	3,753	-	**BMI MD:** 0.10 [95% CI: 0.04, 0.16]; **ALT MD:** 0.65 [95% CI: 0.54, 0.76]; **Elevated ALT OR:** 1.09 [95% CI: 0.95, 1.26]; **AST MD:** 0.55 [95% CI: 0.47, 0.63]; **Elevated AST OR:** 2.41 [95% CI:

First Author	Year	Publication title	DOI	PMID	Study Design	Country	Cohort ID	Cohort description	Genotype	Cohort size (n)	Comp. size (n)	Prevalence statistics	Odds ratio and standardised mean difference statistics
													1.23, 4.71] **ALP MD:**1.80 [95% CI: 1.54, 2.06]; **Elevated ALP OR:** 1.22 [95% CI: 1.07, 1.39]
						GB	d	MZ individuals with high alcohol intake from UK Biobank	MZ	2,688	69,282	-	**BMI MD:** -0.15 [95% CI: -0.37, 0.07]; **ALT MD:** 1.23 [95% CI: 0.55, 1.90; **Elevated ALT OR:** 1.35 [95% CI: 0.87, 2.08]; **AST MD:** 0.40 [95% CI: -0.01, 0.81] **Elevated AST OR:** 1.06 [95% CI: 0.97, 1.34]; **ALP MD:** 2.75 [95% CI: 1.50, 4.00]; **Elevated ALP OR:** 1.37 [95% CI: 0.83, 2.24]
Goltz	2014	Alpha-1 Antitrypsin PiMZ heterozygosity has an independent aggravating effect on liver fibrosis in alcoholic liver disease.	10.1007/s00428-014-1633-3	25070245	Cohort Study Retrospective	DE	-	MZ patients from University of Bonn Medical School	MZ	30	30	-	**ALT MD:** 11.15 [95% CI: -5.23, 27.53]; **AST MD:** 6.92 [95% CI: 0.53, 13.31]; **ALP MD:** 28.80 [95% CI: 7.88, 49.72]
Gupta	2020	Granularity of SERPINA1 alleles by DNA sequencing in CanCOLD	10.1183/13993003.00958-2020	32482783	Cohort study Retrospective	CA	-	MZ patients from Canadian Cohort of Obstructive Lung Disease	MZ	147	1149	Diabetes: 13.56%	**BMI MD:** -0.60 [95% CI: -1.35, 0.15]
Hashemi	2005	High prevalence of Alpha-1 Antitrypsin phenotypes in viral hepatitis B infected patients in Iran.	10.1016/j.hepres.2005.09.035	16260177	Cohort Study Retrospective	IR	-	MZ hepatitis B patients from Tehran Hepatitis Centre and Zahedan Hepatitis Centre	MZ	36	219	-	**Cirrhosis OR:** 2.06 [95% CI: 0.81, 5.22]
Mandorfer	2018	Liver disease in adults with Alpha-1 Antitrypsin deficiency	10.1177/2050640618764057	30083333	Cohort Study Retrospective	AT	-	Heterozygous A1ATD patients from the Medical University of Vienna	Mixed Het.	21	293	-	**Steatosis OR:**0.62 [95% CI: 0.08, 4.81]; **ALT MD:** -4.00 [95% CI: -16.55, 8.55]; **AST MD:** -12.00 [95% CI: -37.17, 13.17]
Molloy	2014	Clarification of the risk of chronic obstructive pulmonary disease in Alpha-1 Antitrypsin deficiency PiMZ heterozygotes.	10.1164/rccm.201311-1984OC	24428606	Cohort Study Retrospective	IE	-	MZ family members of MZ Individuals identified through Irish National AATD Targeted Detection Programme	MZ	89	99	-	**BMI MD:** 0.60 [95% CI: -0.94, 2.14]
Murali	2023	Alpha-1 Antitrypsin Pi*MZ variant increases risk of developing hepatic events in non-alcoholic fatty liver disease patients.	10.1016/j.clinre.2022.102066	36509354	Cohort Study Retrospective	US	-	MZ patients from the University of Iowa Hospitals and Clinics	MZ	162	1249	-	**BMI MD:** 0.70 [95% CI: -0.68, 2.08]
Pons	2021	Utility of Transient Elastography for the Screening of Liver Disease in Patients	10.1111/jth.15556	33923569	Cohort Study Retrospective	ES	-	Heterozygous A1ATD patients from Vall d'Hebron University Hospital, University Complex of Vigo, and Hospital Clínico San Carlos	Mixed Het.	67	81	Diabetes: 2.99% Steatosis: 26.97% Fibrosis: 0.74%	-
Riis	2022	Alpha-1 Antitrypsin Z allele and risk of venous thromboembolism in the general population.	10.1515/cclm-2020-0071	34662507	Cohort Study Retrospective	DK	-	Heterozygous A1ATD individuals from Copenhagen General Population Study	Mixed Het.	5,655	101,365	Diabetes: 4.00%	**BMI MD:** -0.02 [95% CI: -0.07, 0.02]; **ALT MD:** 1.50 [95% CI: 1.42, 1.58]

First Author	Year	Publication title	DOI	PMID	Study Design	Country	Cohort ID	Cohort description	Genotype	Cohort size (n)	Comp. size (n)	Prevalence statistics	Odds ratio and standardised mean difference statistics
Schneider	2020	Liver Phenotypes of European Adults Heterozygous or Homozygous for Pi*Z Variant of AAT (Pi*MZ vs Pi*ZZ genotype) and Noncarriers.	10.1111/jth.15556	32376409	Cohort Prospective	EU	a	MZ individuals from European Alpha-1 Liver Cohort	MZ	419	284	Obesity: 18.00% Steatosis: 29.00% Fibrosis: 14.00%	**BMI MD:** 0.00 [95% CI: -0.75, 0.75]; **Steatosis OR:** 1.10; [95% CI: 0.79, 1.55] **Fibrosis OR:** 3.16 [95% CI: 1.73, 5.78]
						GB	b	MZ individuals from UK Biobank	MZ	17,191	427,310	Diabetes: 4.00%	-
Schuler	2023	Population genetic testing and SERPINA1 sequencing identifies unidentified Alpha-1 Antitrypsin deficiency alleles and gene-environment interaction with hepatitis C infection.	10.1371/journal.pone.0286469	37651384	Cohort Study Retrospective	US	a	MZ individuals with hepatitis C from BioVu, Vanderbilt University's DNA Biobank	MZ	52	1,029	-	**Liver Transplant OR:** 1.96 [95% CI: 1.07, 3.61]
							b	MZ individuals without hepatitis C from BioVu, Vanderbilt University's DNA Biobank	MZ	2,652	492	-	**Liver Transplant OR:** 2.92 [95% CI: 1.06, 8.06]
							c	SZ individuals without hepatitis C from BioVu, Vanderbilt University's DNA Biobank	SZ	139	190	-	**Liver Transplant OR:** 4.98 [95% CI: 1.02, 24.37]
Serban	2022	Unique and shared systemic biomarkers for emphysema in Alpha-1 Antitrypsin LID	10.1016/j.ebiom.2022.104262	36155958	Cohort Study Retrospective	US	-	MZ individuals from the COPDGene Study	MZ	159	5101	-	**BMI MD:** -0.50 [95% CI: -0.48, 1.48]
Strnad	2019	Heterozygous carriage of the Alpha-1 Antitrypsin Pi*Z variant increases the risk to develop liver cirrhosis.	10.1136/gutjnl-2018-316228	30068662	Cohort Study Retrospective	DE & AT	a	MZ patients from multiple hospitals around Germany and Austria	MZ	36	598	-	**Fibrosis OR:** 1.95 [95% CI: 0.99, 3.82]; **Cirrhosis OR:** 3.23 [95% CI: 1.45, 7.20]
						DE & AT	b	Different set of MZ patients from multiple hospitals around Germany and Austria	MZ	22	517	-	**Fibrosis OR:** 1.31 [95% CI: 0.56, 3.09]; **Cirrhosis OR:** 5.25 [95% CI: 1.63, 16.88]
						DE & CH	c	MZ alcohol misusers from multiple hepatology centres around Germany and Switzerland	MZ	75	1590	-	**Cirrhosis OR:** 3.45 [95% CI: 1.99, 5.97]
						DE & CH	d	SZ alcohol misusers from multiple hepatology centres around Germany and Switzerland	SZ	6	1590	-	**Cirrhosis OR:** 13.13 [95% CI: 0.74, 233.49]
						GB	e	MZ alcohol misusers from Royal Free Hospital	MZ	28	571	-	**Cirrhosis OR:** 2.47 [95% CI: 1.10, 5.56]

First Author	Year	Publication title	DOI	PMID	Study Design	Country	Cohort ID	Cohort description	Genotype	Cohort size (n)	Comp. size (n)	Prevalence statistics	Odds ratio and standardised mean difference statistics
Tanash	2015	The Swedish α1-Antitrypsin Screening Study: Health Status and Lung and Liver Function at Age 34	10.1513/AnnalsATS.201410-452OC	25803183	Cohort Stude Prospective	SE	-	SZ individuals identified from Swedish Neonatal Screening	SZ	48	239	-	**ALT MD:** 5.90 [95% CI: 1.27, 10.53];<br>**AST MD:** 1.30 [95% CI: 0.80, 1.80]
Thun	2012	SERPINA1 PiZ and PiS heterozygotes and lung function decline in the SAPALDIA cohort.	10.1371/journal.pone.0042728	22912729	Cohort Study Prospective	CH	-	MZ individuals from the Swiss Cohort Study on Air Pollution and Lung and Heart Diseases in Adults (SAPALDIA)	MZ	112	4,207	-	**BMI MD:** -0.23 [95% CI: -0.42, -0.03]
