# Supplementary material for: Meta‐Analysis: Redefining Liver Disease Risk in Heterozygous Alpha‐1 Antitrypsin Deficiency
Source: Aliment Pharmacol Ther. 2026 Jul 12;64(4):430–40. doi: 10.1111/apt.70814 (PMC13419271; doi:10.1111/apt.70814)
Supplement: Supplementary file 1 — Table S1: Search terms used in the systematic review. Table S2: Characteristics of included studies. Table S3: PRISMA checklist. Figure S1: Study selection and characteristics of included cohorts. (A) PRISMA flow diagram summarizing study identification, screening, eligibility assessment, and inclusion. (B) Cumulative number of eligible publications by year. (C) Sex distribution across SERPINA1 genotype groups, with random‐effects meta‐regression showing no significant association between female proportion and genotype. (D) Geographic distribution of included cohorts by country or region. Figure S2: Study quality assessment using the Newcastle–Ottawa Scale. Non‐randomized studies were scored from 0 to 9 across the domains of selection, comparability, and outcome. Total scores were categorized as high (7–9), fair (4–6), or low (0–3) quality. Figure S3: Leave‐one‐out sensitivity analysis. Panels show the impact of excluding individual studies on pooled estimates for (A) comorbidities associated with metabolic syndrome (obesity, type 2 diabetes, steatosis), (B) serum liver enzymes (ALT, AST, ALP), and (C) liver disease outcomes (fibrosis, cirrhosis, liver transplantation). Each point represents the pooled estimate recalculated after omitting the indicated study. The green band represents the 95% CI of the complete meta‐analysis. Labels indicate whether the leave‐one‐out pooled estimate differed from the complete‐set pooled estimate using a two‐sided z test. Exclusion of any single study does not materially change the summary estimates for any outcome, ns = p > 0.05, * = p < 0.05. Figure S4: Sensitivity analysis of metabolic comorbidities and hepatic steatosis stratified by SERPINA1 genotype. Pooled estimates are presented by genotype subgroup for obesity prevalence (A), BMI mean difference versus MM controls (B), type 2 diabetes prevalence (C), steatosis prevalence (D), and steatosis odds ratio versus MM controls (E). Subgroup estimates are shown for MZ, SZ, and combin [file APT-64-430-s001.zip › apt70814-sup-0001-Supinfo01/Supplementary Table S3.pdf]

PRISMA 2020 Checklist

Section and Topic	Item #	Checklist item	Location where item is reported
**TITLE**			
Title	1	Identify the report as a systematic review.	Line 44
**ABSTRACT**			
Abstract	2	See the PRISMA 2020 for Abstracts checklist.	Lines 37-61
**INTRODUCTION**			
Rationale	3	Describe the rationale for the review in the context of existing knowledge.	Lines 105-131
Objectives	4	Provide an explicit statement of the objective(s) or question(s) the review addresses.	Lines 132-136
**METHODS**			
Eligibility criteria	5	Specify the inclusion and exclusion criteria for the review and how studies were grouped for the syntheses.	Lines 152-157
Information sources	6	Specify all databases, registers, websites, organisations, reference lists and other sources searched or consulted to identify studies. Specify the date when each source was last searched or consulted.	Lines 141-151
Search strategy	7	Present the full search strategies for all databases, registers and websites, including any filters and limits used.	Supplementary Table T1
Selection process	8	Specify the methods used to decide whether a study met the inclusion criteria of the review, including how many reviewers screened each record and each report retrieved, whether they worked independently, and if applicable, details of automation tools used in the process.	Lines 168-175
Data collection process	9	Specify the methods used to collect data from reports, including how many reviewers collected data from each report, whether they worked independently, any processes for obtaining or confirming data from study investigators, and if applicable, details of automation tools used in the process.	Lines 177-182
Data items	10a	List and define all outcomes for which data were sought. Specify whether all results that were compatible with each outcome domain in each study were sought (e.g. for all measures, time points, analyses), and if not, the methods used to decide which results to collect.	Lines 191-205
	10b	List and define all other variables for which data were sought (e.g. participant and intervention characteristics, funding sources). Describe any assumptions made about any missing or unclear information.	Lines 191-205
Study risk of bias assessment	11	Specify the methods used to assess risk of bias in the included studies, including details of the tool(s) used, how many reviewers assessed each study and whether they worked independently, and if applicable, details of automation tools used in the process.	Lines 185-190
Effect measures	12	Specify for each outcome the effect measure(s) (e.g. risk ratio, mean difference) used in the synthesis or presentation of results.	Lines 204-216
Synthesis methods	13a	Describe the processes used to decide which studies were eligible for each synthesis (e.g. tabulating the study intervention characteristics and comparing against the planned groups for each synthesis (item #5)).	Lines 205-216
	13b	Describe any methods required to prepare the data for presentation or synthesis, such as handling of missing summary statistics, or data conversions.	Lines 206-214
	13c	Describe any methods used to tabulate or visually display results of individual studies and syntheses.	Lines 205-216
	13d	Describe any methods used to synthesize results and provide a rationale for the choice(s). If meta-analysis was performed, describe the model(s), method(s) to identify the presence and extent of statistical heterogeneity, and software package(s) used.	Lines 206-237
	13e	Describe any methods used to explore possible causes of heterogeneity among study results (e.g. subgroup analysis, meta-regression).	Lines 215-237
	13f	Describe any sensitivity analyses conducted to assess robustness of the synthesized results.	Lines 215-237
Reporting bias assessment	14	Describe any methods used to assess risk of bias due to missing results in a synthesis (arising from reporting biases).	Lines 215-237
Certainty	15	Describe any methods used to assess certainty (or confidence) in the body of evidence for an outcome.	Lines 215-237

Section and Topic	Item #	Checklist item	Location where item is reported
assessment			
**RESULTS**			
Study selection	16a	Describe the results of the search and selection process, from the number of records identified in the search to the number of studies included in the review, ideally using a flow diagram.	Lines 240-242, Supplementary Figure S1A
	16b	Cite studies that might appear to meet the inclusion criteria, but which were excluded, and explain why they were excluded.	Supplementary Figure S1A
Study characteristics	17	Cite each included study and present its characteristics.	Supplementary Table T2
Risk of bias in studies	18	Present assessments of risk of bias for each included study.	Lines 253-256, Supplementary Figure S8
Results of individual studies	19	For all outcomes, present, for each study: (a) summary statistics for each group (where appropriate) and (b) an effect estimate and its precision (e.g. confidence/credible interval), ideally using structured tables or plots.	Figures 1-3
Results of syntheses	20a	For each synthesis, briefly summarise the characteristics and risk of bias among contributing studies.	Supplementary Figures S3, S8
	20b	Present results of all statistical syntheses conducted. If meta-analysis was done, present for each the summary estimate and its precision (e.g. confidence/credible interval) and measures of statistical heterogeneity. If comparing groups, describe the direction of the effect.	Figures 1-3
	20c	Present results of all investigations of possible causes of heterogeneity among study results.	Lines 259-261, Supplementary Figure S3-S7
	20d	Present results of all sensitivity analyses conducted to assess the robustness of the synthesized results.	Lines 259-261, Supplementary Figure S3-S7
Reporting biases	21	Present assessments of risk of bias due to missing results (arising from reporting biases) for each synthesis assessed.	Lines 259-261, Supplementary Figure S8
Certainty of evidence	22	Present assessments of certainty (or confidence) in the body of evidence for each outcome assessed.	Lines<br>265-267, 269-271, 277-285, 290-297
**DISCUSSION**			
Discussion	23a	Provide a general interpretation of the results in the context of other evidence.	Lines 300-316
	23b	Discuss any limitations of the evidence included in the review.	Lines 385-394
	23c	Discuss any limitations of the review processes used.	Lines 385-394
	23d	Discuss implications of the results for practice, policy, and future research.	Lines 395-412
**OTHER INFORMATION**			

PRISMA 2020 Checklist

Section and Topic	Item #	Checklist item	Location where item is reported
Registration and protocol	24a	Provide registration information for the review, including register name and registration number, or state that the review was not registered.	Line 167
	24b	Indicate where the review protocol can be accessed, or state that a protocol was not prepared.	Line 167
	24c	Describe and explain any amendments to information provided at registration or in the protocol.	-
Support	25	Describe sources of financial or non-financial support for the review, and the role of the funders or sponsors in the review.	Line 23
Competing interests	26	Declare any competing interests of review authors.	Line 22
Availability of data, code and other materials	27	Report which of the following are publicly available and where they can be found: template data collection forms; data extracted from included studies; data used for all analyses; analytic code; any other materials used in the review.	Supplementary Table T2

From: Page MJ, McKenzie JE, Bossuyt PM, Boutron I, Hoffmann TC, Mulrow CD, et al. The PRISMA 2020 statement: an updated guideline for reporting systematic reviews. BMJ 2021;372:n71. doi: 10.1136/bmj.n71.
This work is licensed under CC BY 4.0. To view a copy of this license, visit https://creativecommons.org/licenses/by/4.0/
