# Supplementary figures and images for: Meta‐Analysis: Redefining Liver Disease Risk in Heterozygous Alpha‐1 Antitrypsin Deficiency
Source: Aliment Pharmacol Ther. 2026 Jul 12;64(4):430–40. doi: 10.1111/apt.70814 (PMC13419271; doi:10.1111/apt.70814)

# Supplementary Figure S1

**A**

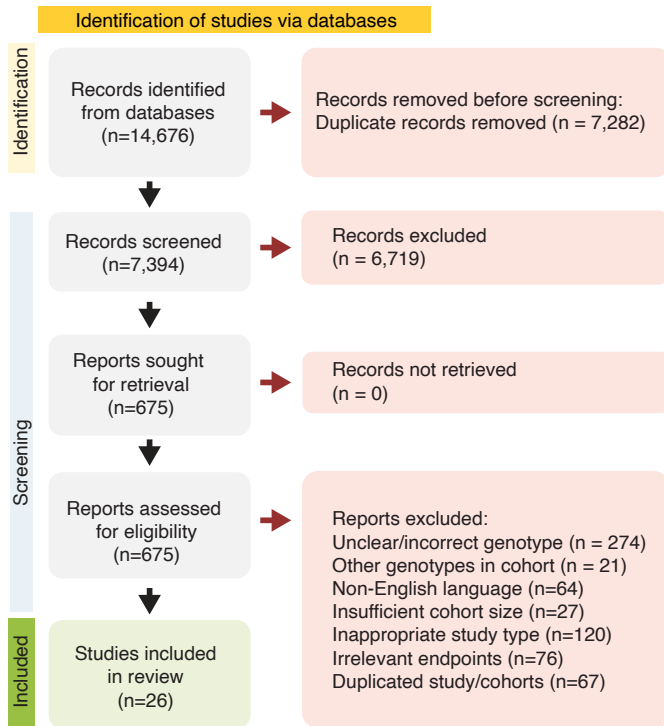

**B**

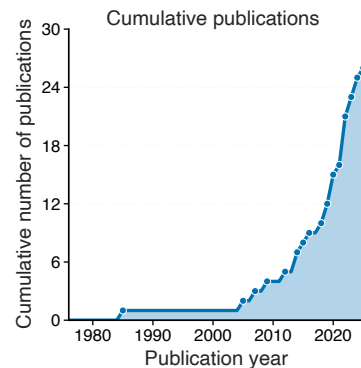

**C**

**Gender distribution per genotype**

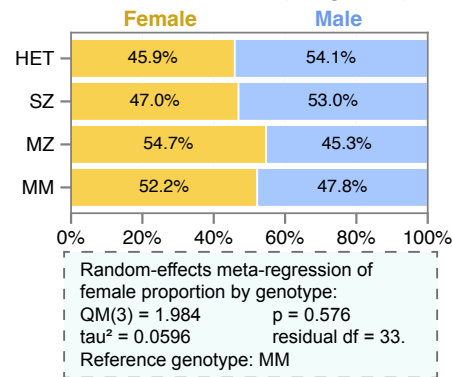

**D**

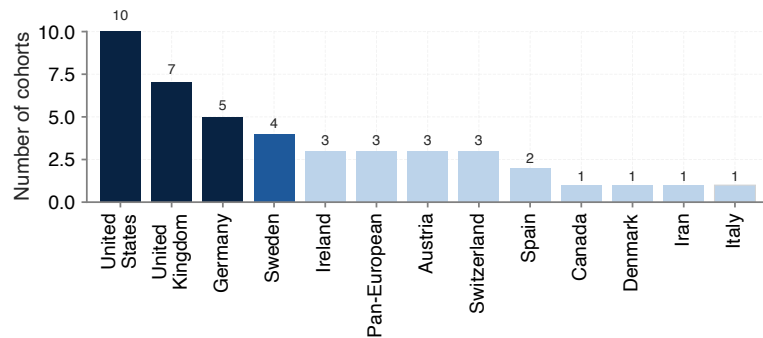

Supplement: Supplementary file 1 — Table S1: Search terms used in the systematic review. Table S2: Characteristics of included studies. Table S3: PRISMA checklist. Figure S1: Study selection and characteristics of included cohorts. (A) PRISMA flow diagram summarizing study identification, screening, eligibility assessment, and inclusion. (B) Cumulative number of eligible publications by year. (C) Sex distribution across SERPINA1 genotype groups, with random‐effects meta‐regression showing no significant association between female proportion and genotype. (D) Geographic distribution of included cohorts by country or region. Figure S2: Study quality assessment using the Newcastle–Ottawa Scale. Non‐randomized studies were scored from 0 to 9 across the domains of selection, comparability, and outcome. Total scores were categorized as high (7–9), fair (4–6), or low (0–3) quality. Figure S3: Leave‐one‐out sensitivity analysis. Panels show the impact of excluding individual studies on pooled estimates for (A) comorbidities associated with metabolic syndrome (obesity, type 2 diabetes, steatosis), (B) serum liver enzymes (ALT, AST, ALP), and (C) liver disease outcomes (fibrosis, cirrhosis, liver transplantation). Each point represents the pooled estimate recalculated after omitting the indicated study. The green band represents the 95% CI of the complete meta‐analysis. Labels indicate whether the leave‐one‐out pooled estimate differed from the complete‐set pooled estimate using a two‐sided z test. Exclusion of any single study does not materially change the summary estimates for any outcome, ns = p > 0.05, * = p < 0.05. Figure S4: Sensitivity analysis of metabolic comorbidities and hepatic steatosis stratified by SERPINA1 genotype. Pooled estimates are presented by genotype subgroup for obesity prevalence (A), BMI mean difference versus MM controls (B), type 2 diabetes prevalence (C), steatosis prevalence (D), and steatosis odds ratio versus MM controls (E). Subgroup estimates are shown for MZ, SZ, and combin [file APT-64-430-s001.zip › apt70814-sup-0001-Supinfo01/Supplementary Figure S1.pdf]

# Supplementary Figure S3

A

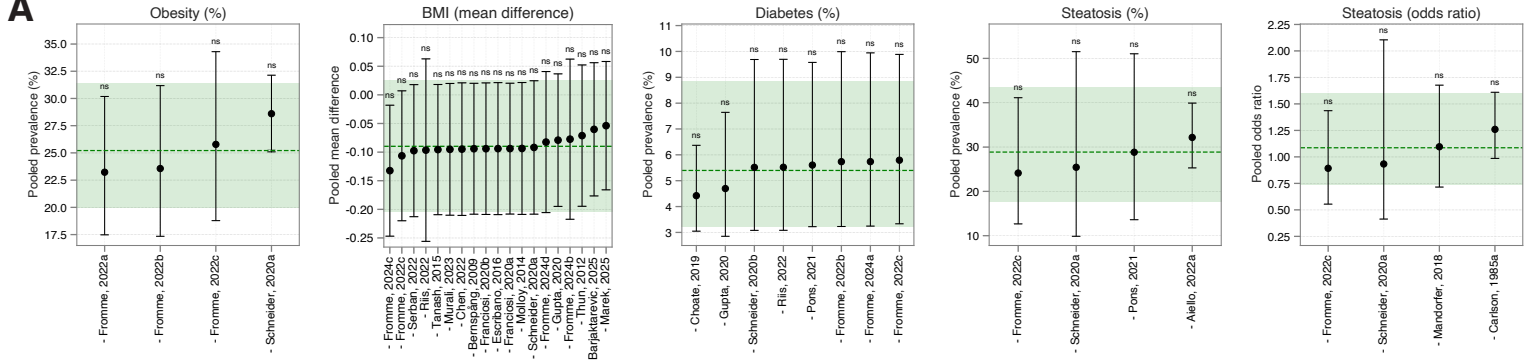

B

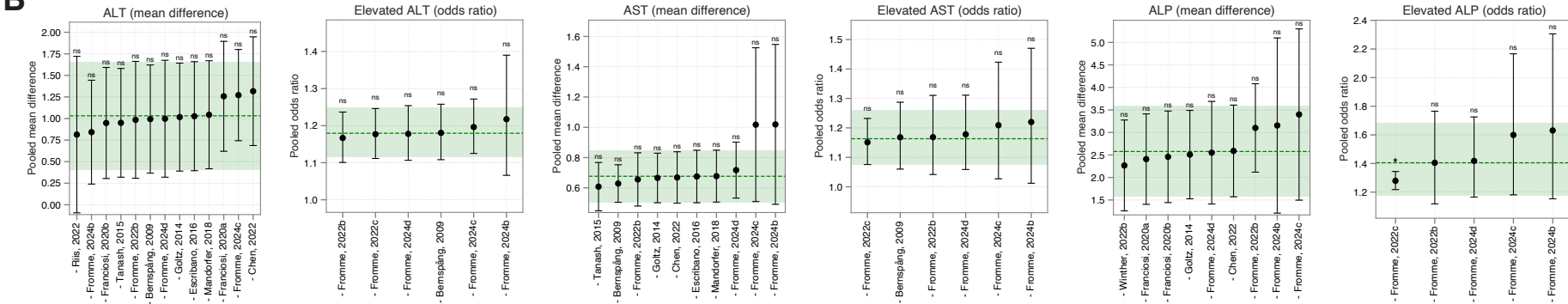

C

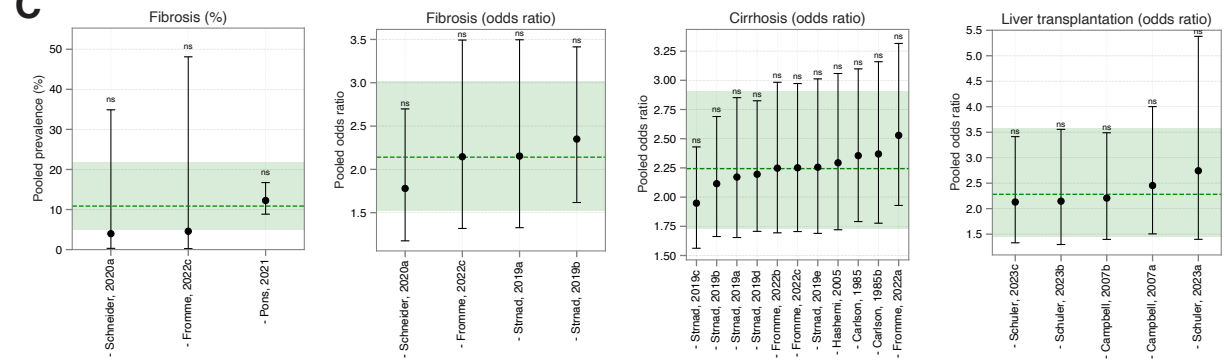

Supplement: Supplementary file 1 — Table S1: Search terms used in the systematic review. Table S2: Characteristics of included studies. Table S3: PRISMA checklist. Figure S1: Study selection and characteristics of included cohorts. (A) PRISMA flow diagram summarizing study identification, screening, eligibility assessment, and inclusion. (B) Cumulative number of eligible publications by year. (C) Sex distribution across SERPINA1 genotype groups, with random‐effects meta‐regression showing no significant association between female proportion and genotype. (D) Geographic distribution of included cohorts by country or region. Figure S2: Study quality assessment using the Newcastle–Ottawa Scale. Non‐randomized studies were scored from 0 to 9 across the domains of selection, comparability, and outcome. Total scores were categorized as high (7–9), fair (4–6), or low (0–3) quality. Figure S3: Leave‐one‐out sensitivity analysis. Panels show the impact of excluding individual studies on pooled estimates for (A) comorbidities associated with metabolic syndrome (obesity, type 2 diabetes, steatosis), (B) serum liver enzymes (ALT, AST, ALP), and (C) liver disease outcomes (fibrosis, cirrhosis, liver transplantation). Each point represents the pooled estimate recalculated after omitting the indicated study. The green band represents the 95% CI of the complete meta‐analysis. Labels indicate whether the leave‐one‐out pooled estimate differed from the complete‐set pooled estimate using a two‐sided z test. Exclusion of any single study does not materially change the summary estimates for any outcome, ns = p > 0.05, * = p < 0.05. Figure S4: Sensitivity analysis of metabolic comorbidities and hepatic steatosis stratified by SERPINA1 genotype. Pooled estimates are presented by genotype subgroup for obesity prevalence (A), BMI mean difference versus MM controls (B), type 2 diabetes prevalence (C), steatosis prevalence (D), and steatosis odds ratio versus MM controls (E). Subgroup estimates are shown for MZ, SZ, and combin [file APT-64-430-s001.zip › apt70814-sup-0001-Supinfo01/Supplementary Figure S3.pdf]

Supplementary Figure 7

A

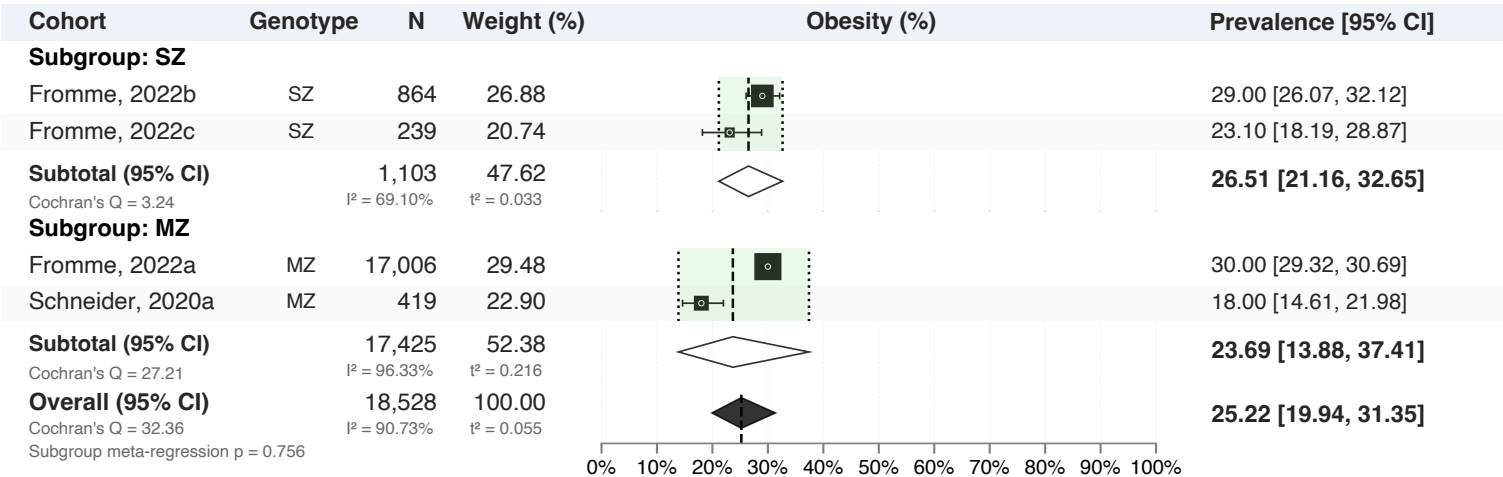

B

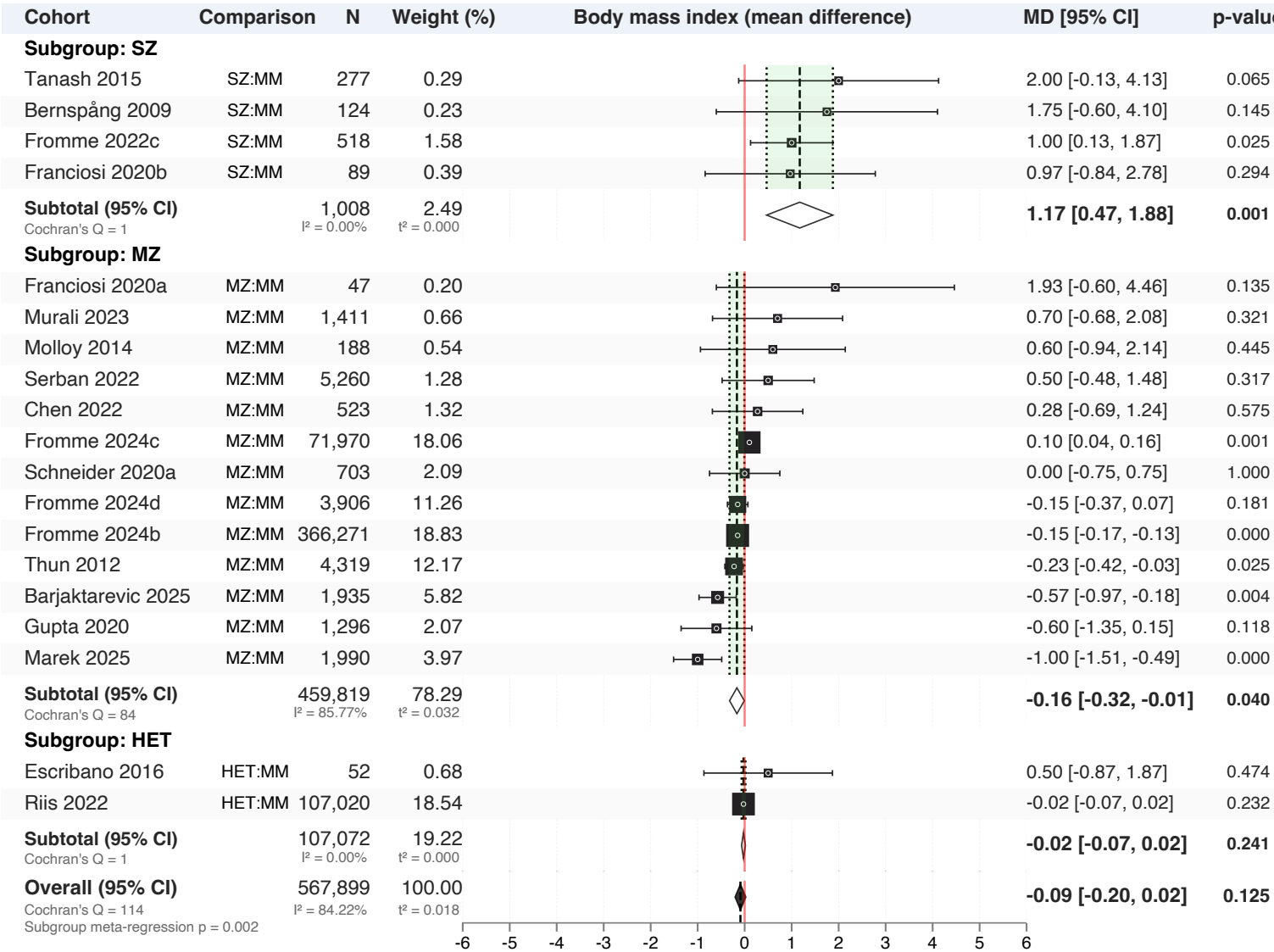

C

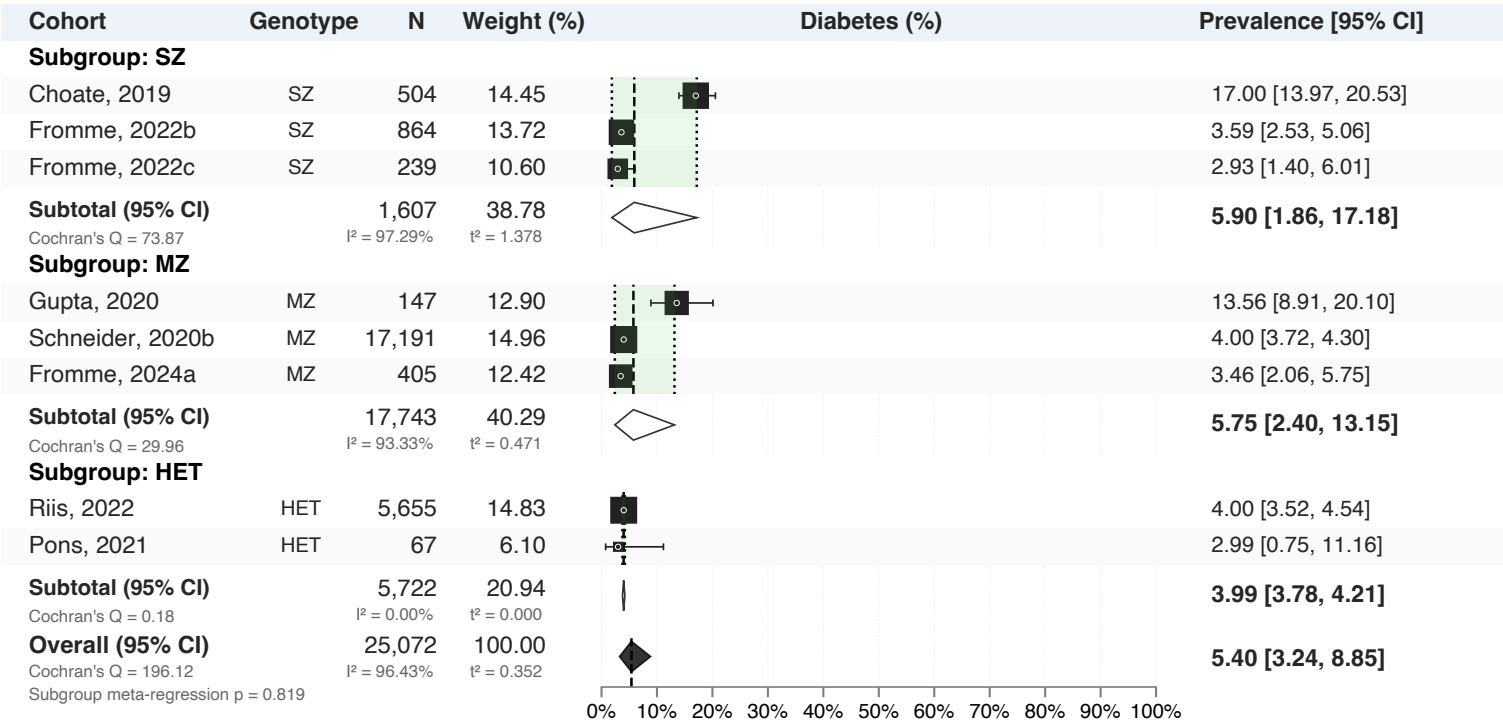

D

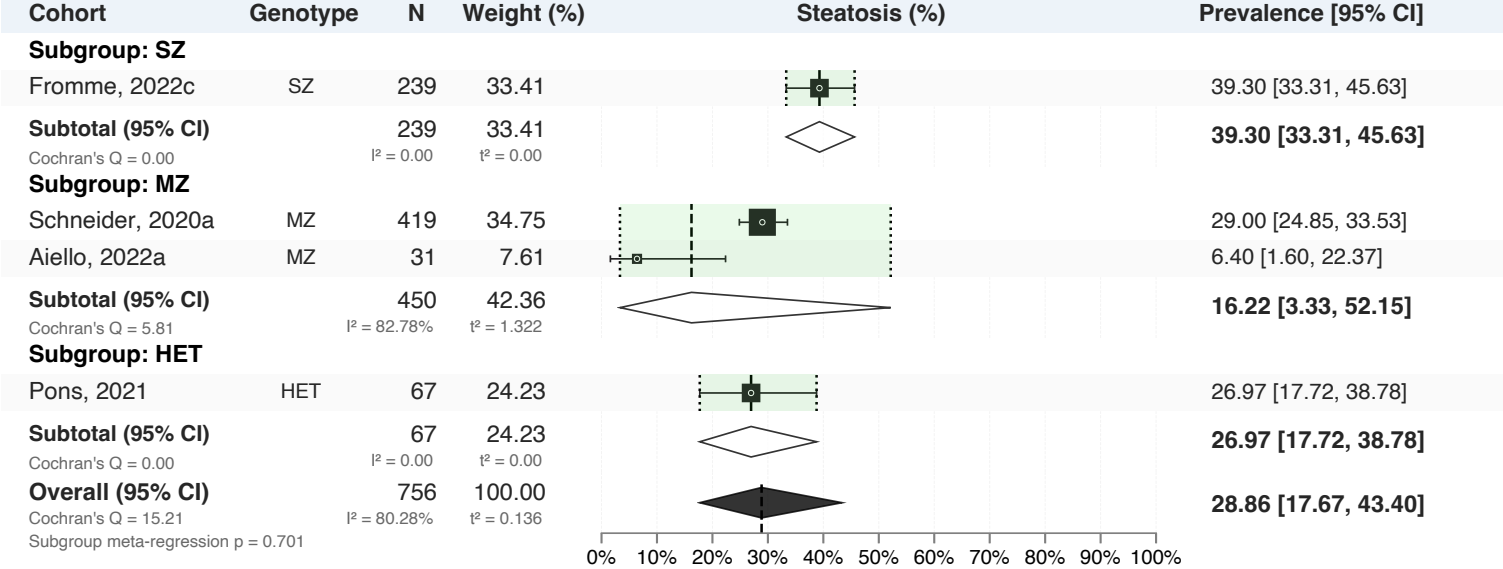

E

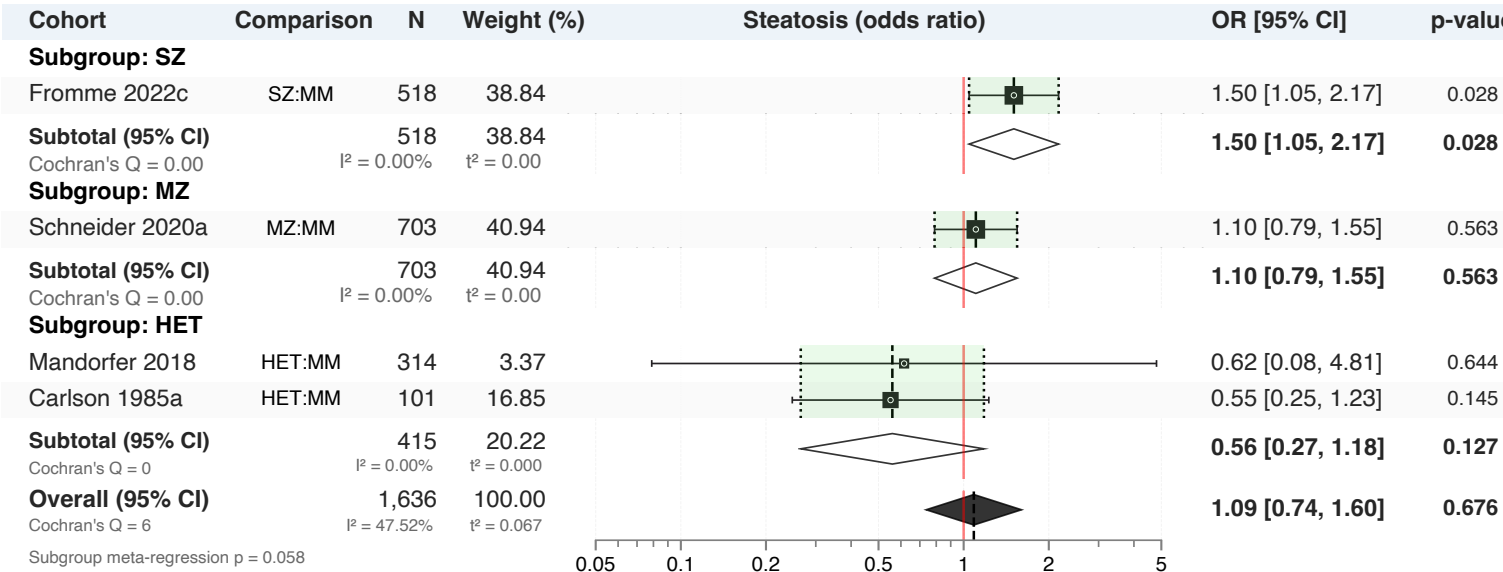

Supplement: Supplementary file 1 — Table S1: Search terms used in the systematic review. Table S2: Characteristics of included studies. Table S3: PRISMA checklist. Figure S1: Study selection and characteristics of included cohorts. (A) PRISMA flow diagram summarizing study identification, screening, eligibility assessment, and inclusion. (B) Cumulative number of eligible publications by year. (C) Sex distribution across SERPINA1 genotype groups, with random‐effects meta‐regression showing no significant association between female proportion and genotype. (D) Geographic distribution of included cohorts by country or region. Figure S2: Study quality assessment using the Newcastle–Ottawa Scale. Non‐randomized studies were scored from 0 to 9 across the domains of selection, comparability, and outcome. Total scores were categorized as high (7–9), fair (4–6), or low (0–3) quality. Figure S3: Leave‐one‐out sensitivity analysis. Panels show the impact of excluding individual studies on pooled estimates for (A) comorbidities associated with metabolic syndrome (obesity, type 2 diabetes, steatosis), (B) serum liver enzymes (ALT, AST, ALP), and (C) liver disease outcomes (fibrosis, cirrhosis, liver transplantation). Each point represents the pooled estimate recalculated after omitting the indicated study. The green band represents the 95% CI of the complete meta‐analysis. Labels indicate whether the leave‐one‐out pooled estimate differed from the complete‐set pooled estimate using a two‐sided z test. Exclusion of any single study does not materially change the summary estimates for any outcome, ns = p > 0.05, * = p < 0.05. Figure S4: Sensitivity analysis of metabolic comorbidities and hepatic steatosis stratified by SERPINA1 genotype. Pooled estimates are presented by genotype subgroup for obesity prevalence (A), BMI mean difference versus MM controls (B), type 2 diabetes prevalence (C), steatosis prevalence (D), and steatosis odds ratio versus MM controls (E). Subgroup estimates are shown for MZ, SZ, and combin [file APT-64-430-s001.zip › apt70814-sup-0001-Supinfo01/Supplementary Figure S4.pdf]

Supplementary Figure S5

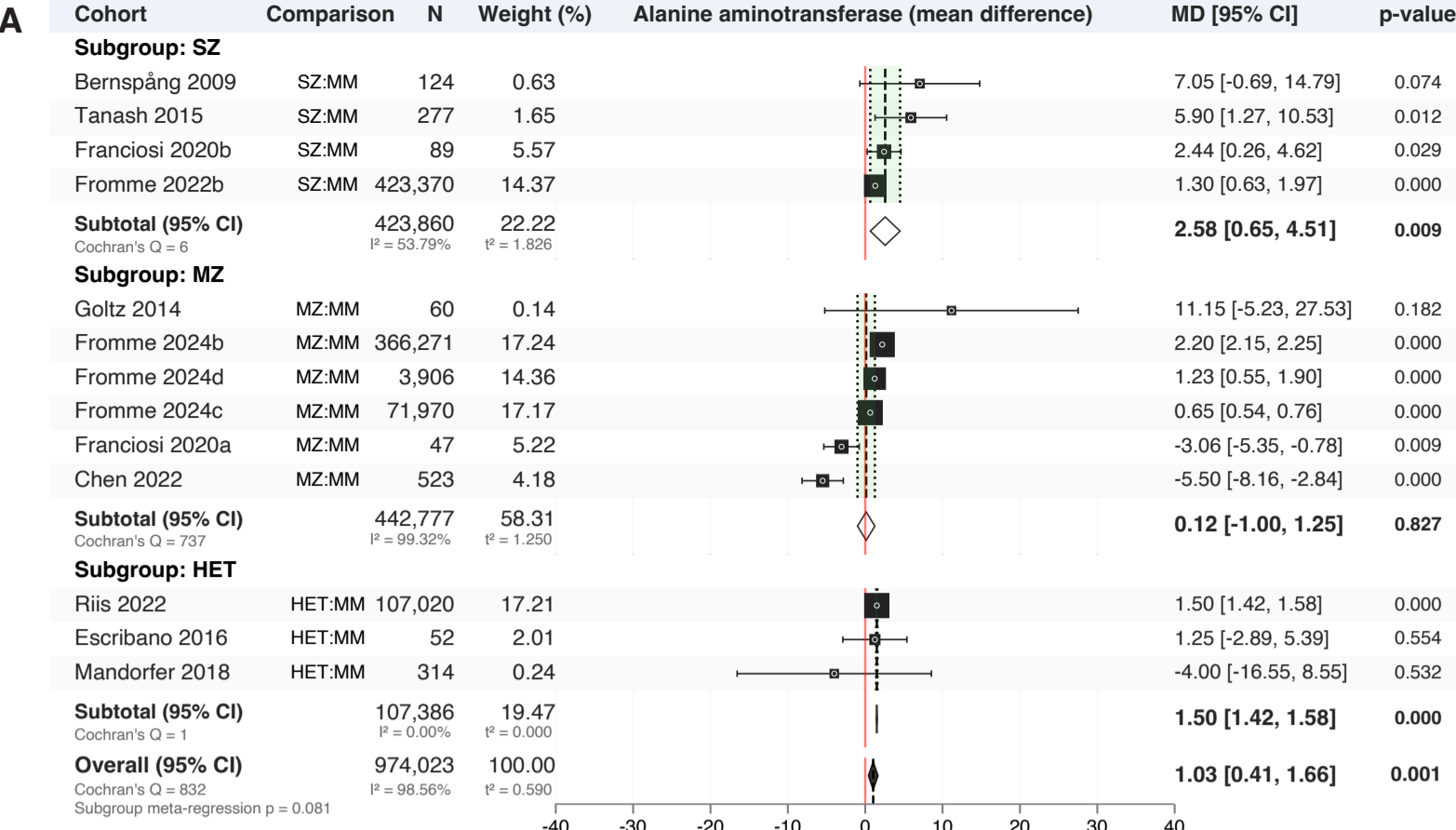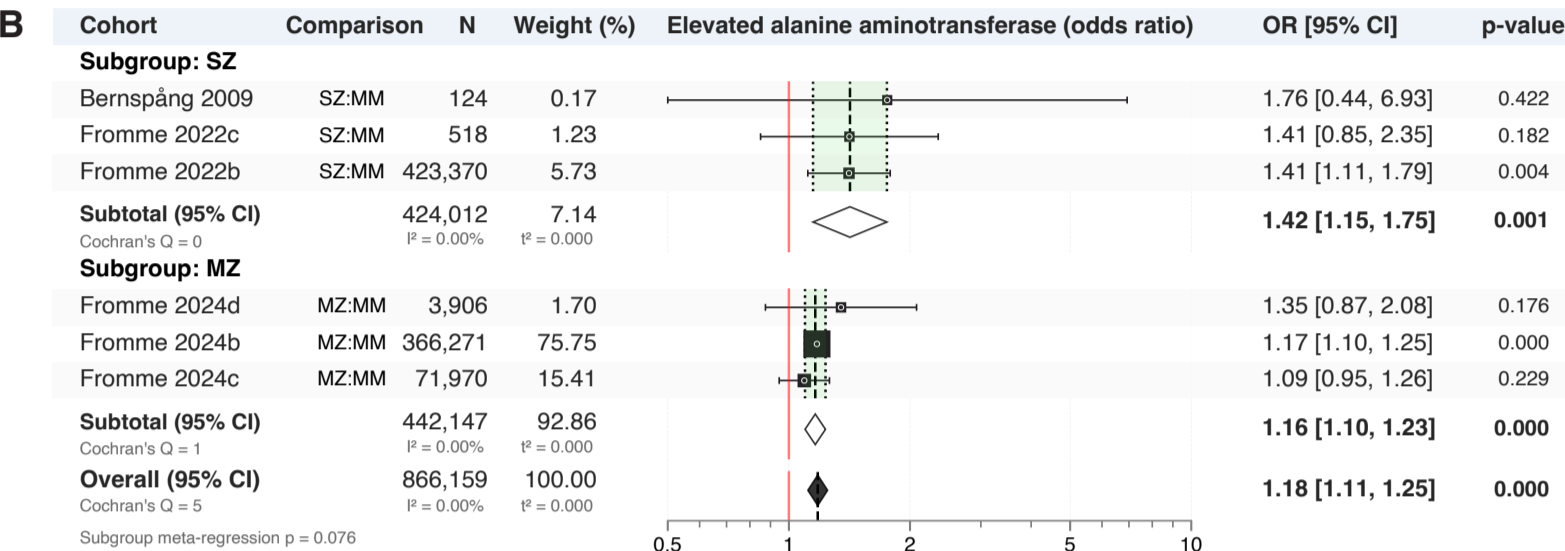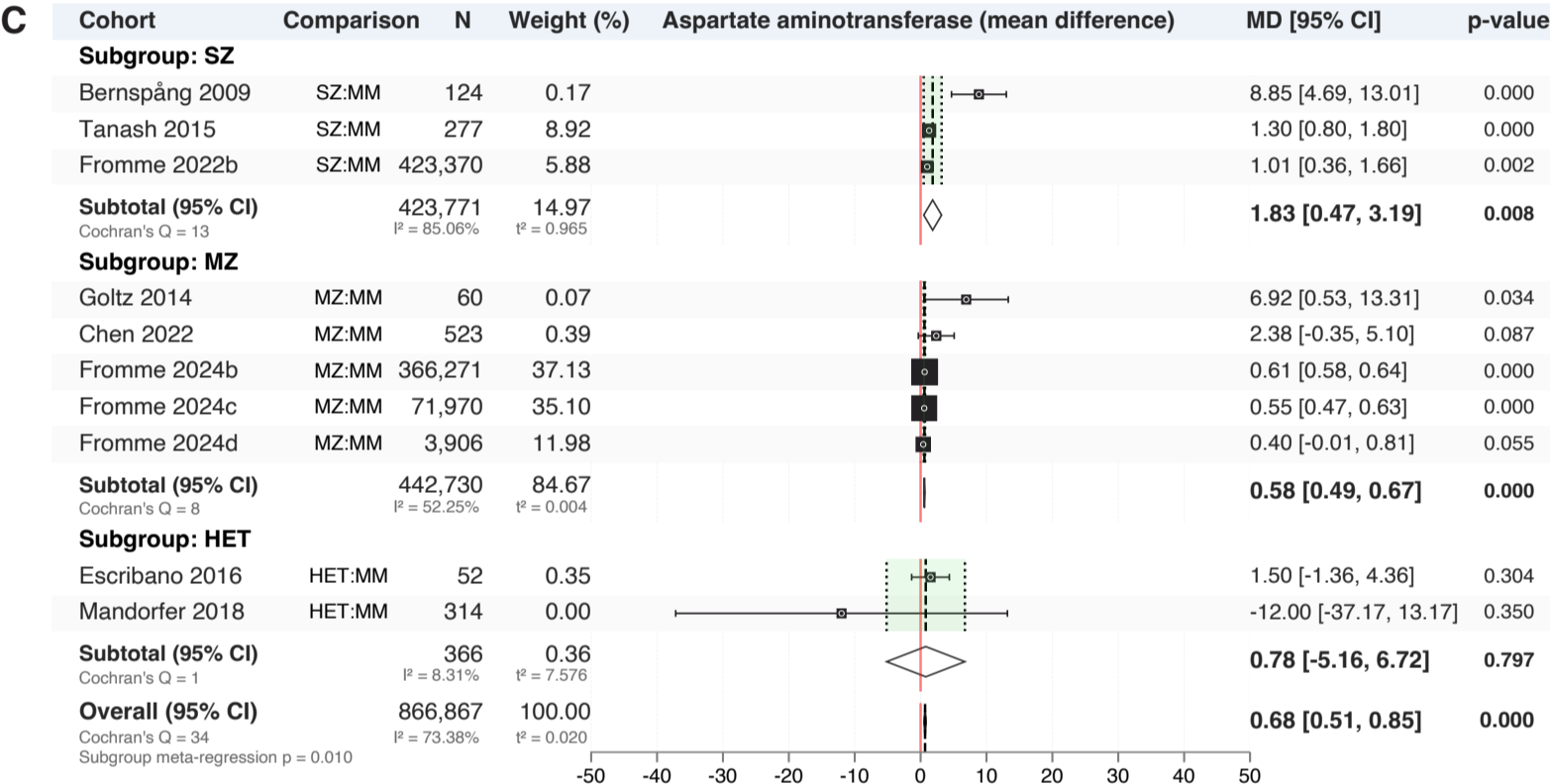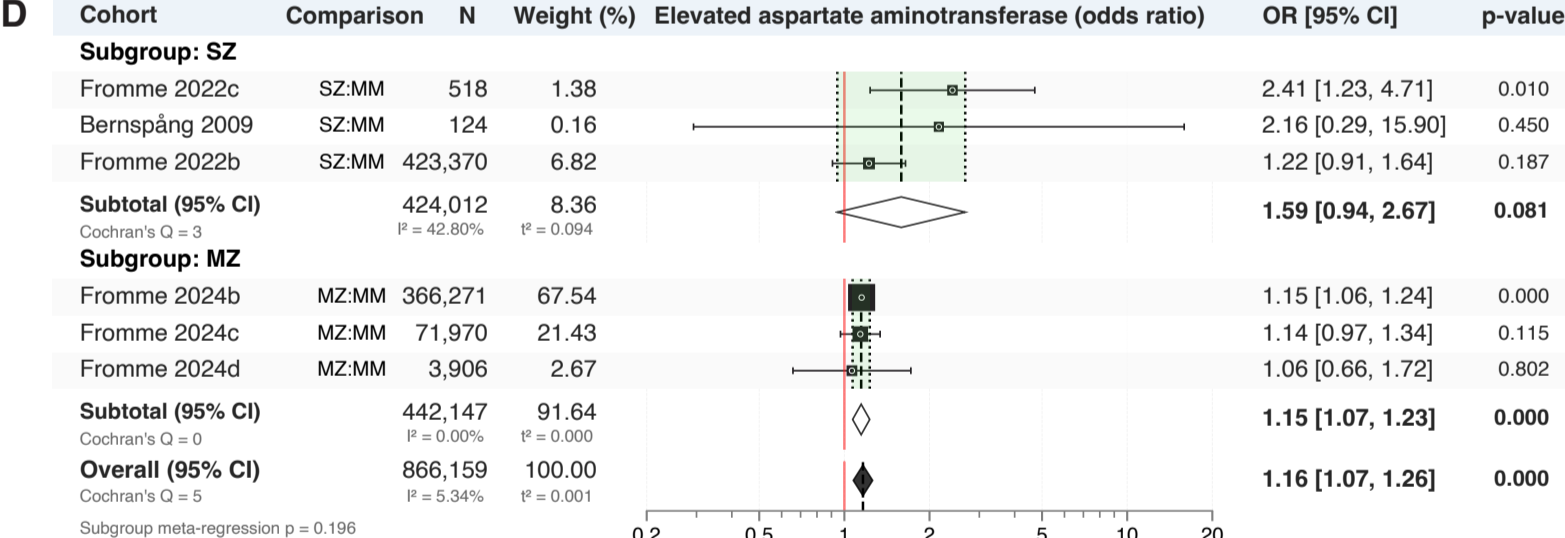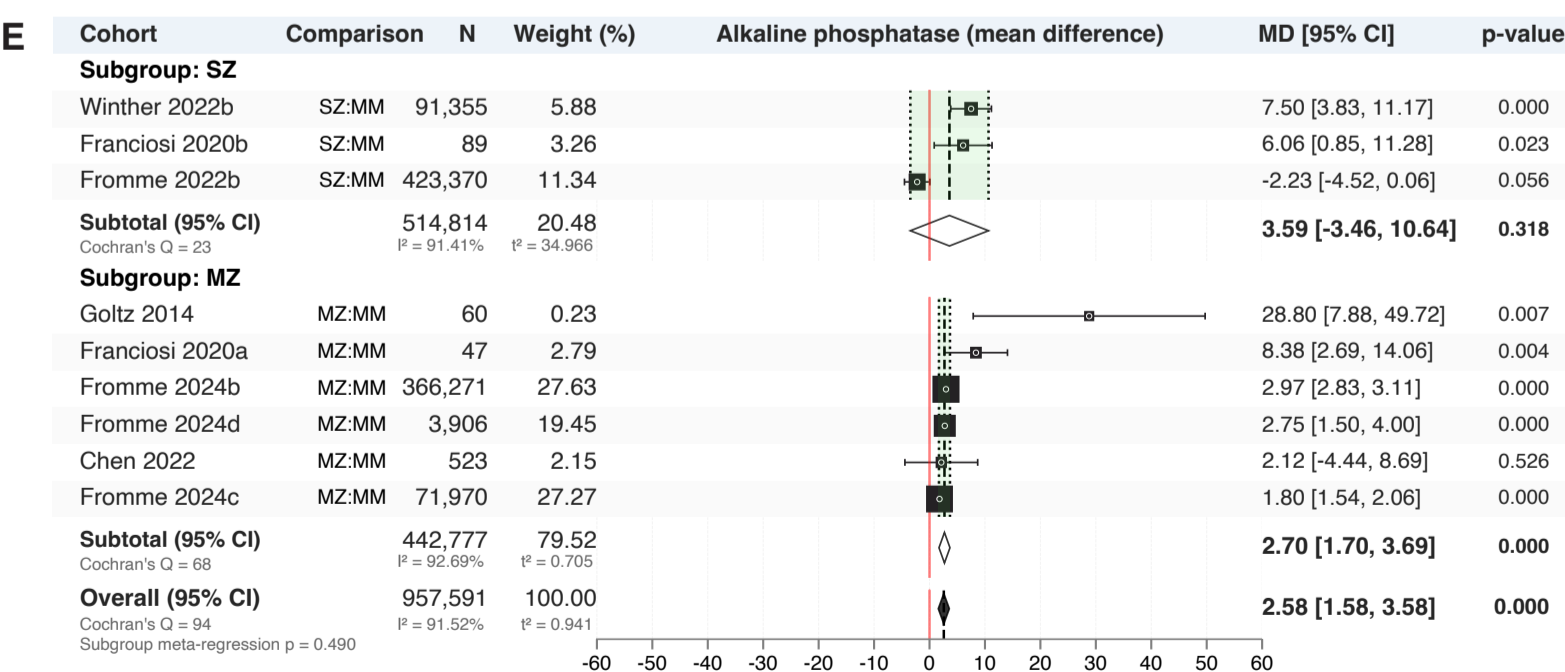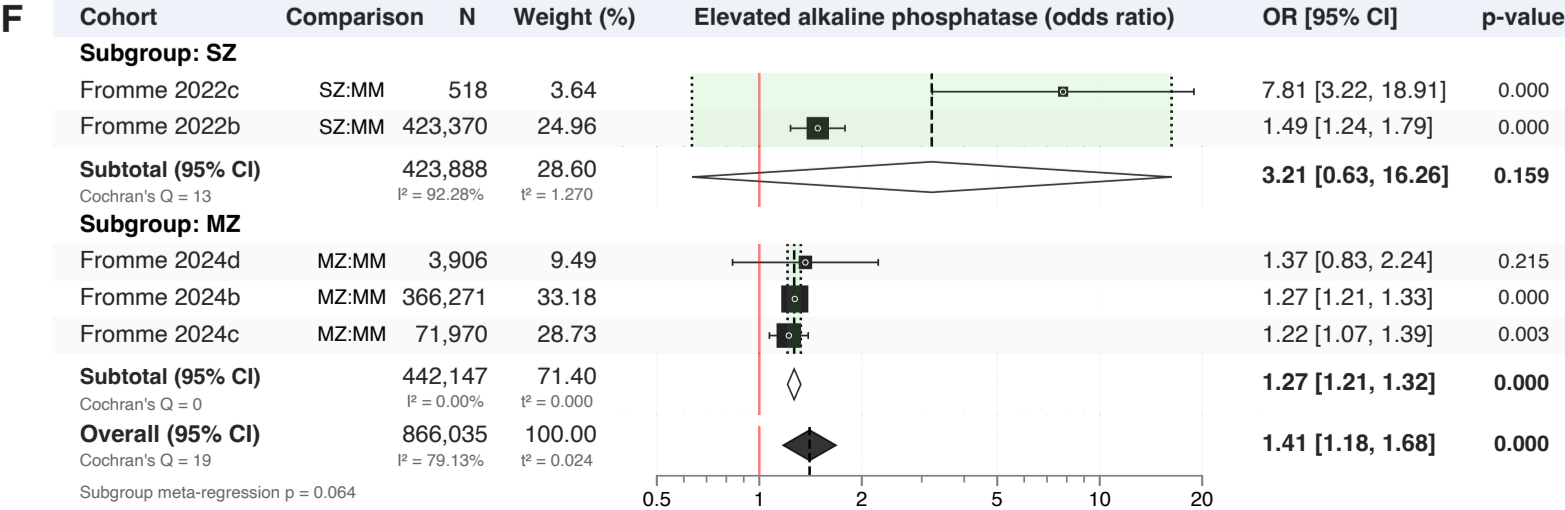

Supplement: Supplementary file 1 — Table S1: Search terms used in the systematic review. Table S2: Characteristics of included studies. Table S3: PRISMA checklist. Figure S1: Study selection and characteristics of included cohorts. (A) PRISMA flow diagram summarizing study identification, screening, eligibility assessment, and inclusion. (B) Cumulative number of eligible publications by year. (C) Sex distribution across SERPINA1 genotype groups, with random‐effects meta‐regression showing no significant association between female proportion and genotype. (D) Geographic distribution of included cohorts by country or region. Figure S2: Study quality assessment using the Newcastle–Ottawa Scale. Non‐randomized studies were scored from 0 to 9 across the domains of selection, comparability, and outcome. Total scores were categorized as high (7–9), fair (4–6), or low (0–3) quality. Figure S3: Leave‐one‐out sensitivity analysis. Panels show the impact of excluding individual studies on pooled estimates for (A) comorbidities associated with metabolic syndrome (obesity, type 2 diabetes, steatosis), (B) serum liver enzymes (ALT, AST, ALP), and (C) liver disease outcomes (fibrosis, cirrhosis, liver transplantation). Each point represents the pooled estimate recalculated after omitting the indicated study. The green band represents the 95% CI of the complete meta‐analysis. Labels indicate whether the leave‐one‐out pooled estimate differed from the complete‐set pooled estimate using a two‐sided z test. Exclusion of any single study does not materially change the summary estimates for any outcome, ns = p > 0.05, * = p < 0.05. Figure S4: Sensitivity analysis of metabolic comorbidities and hepatic steatosis stratified by SERPINA1 genotype. Pooled estimates are presented by genotype subgroup for obesity prevalence (A), BMI mean difference versus MM controls (B), type 2 diabetes prevalence (C), steatosis prevalence (D), and steatosis odds ratio versus MM controls (E). Subgroup estimates are shown for MZ, SZ, and combin [file APT-64-430-s001.zip › apt70814-sup-0001-Supinfo01/Supplementary Figure S5.pdf]

Supplementary Figure S6

A

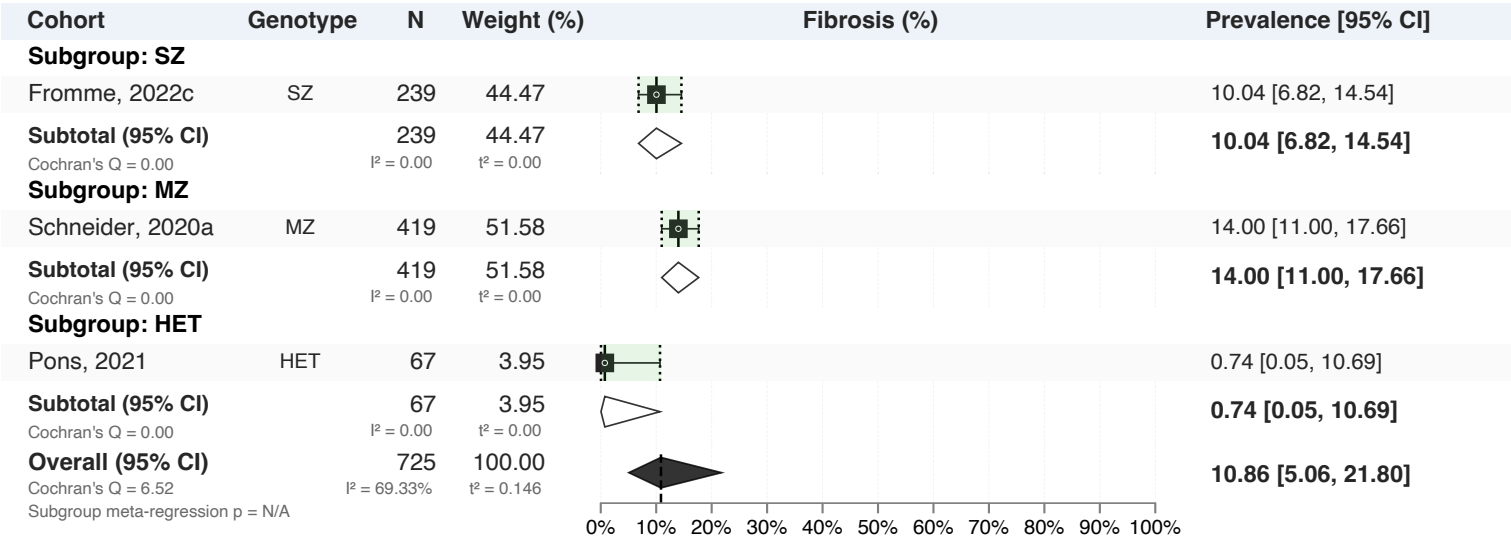

B

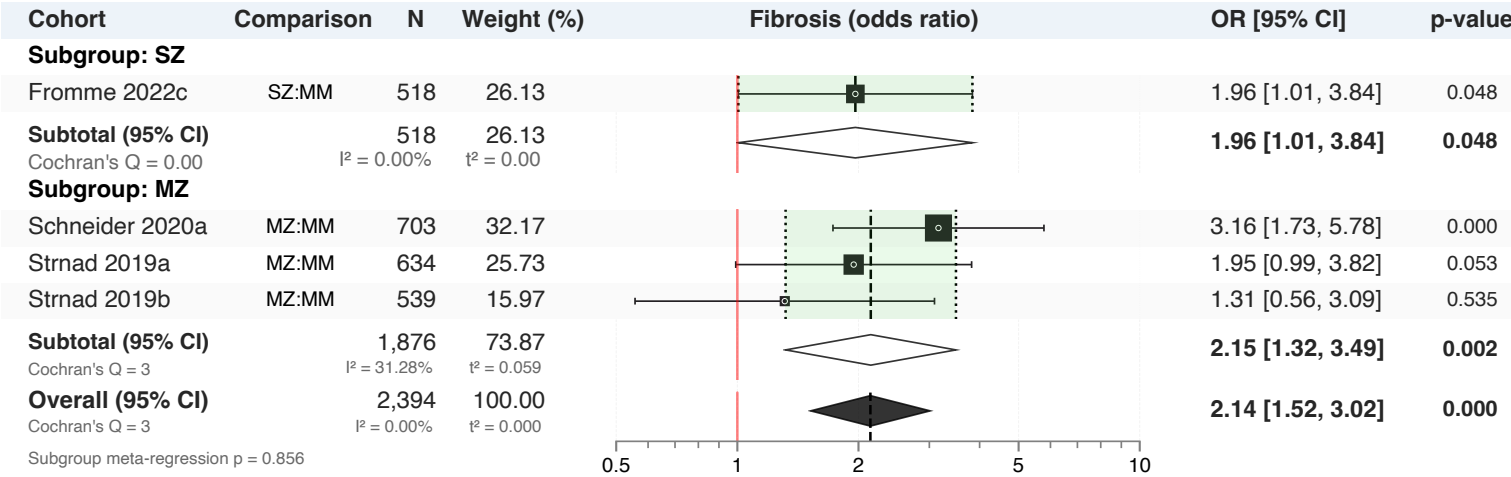

C

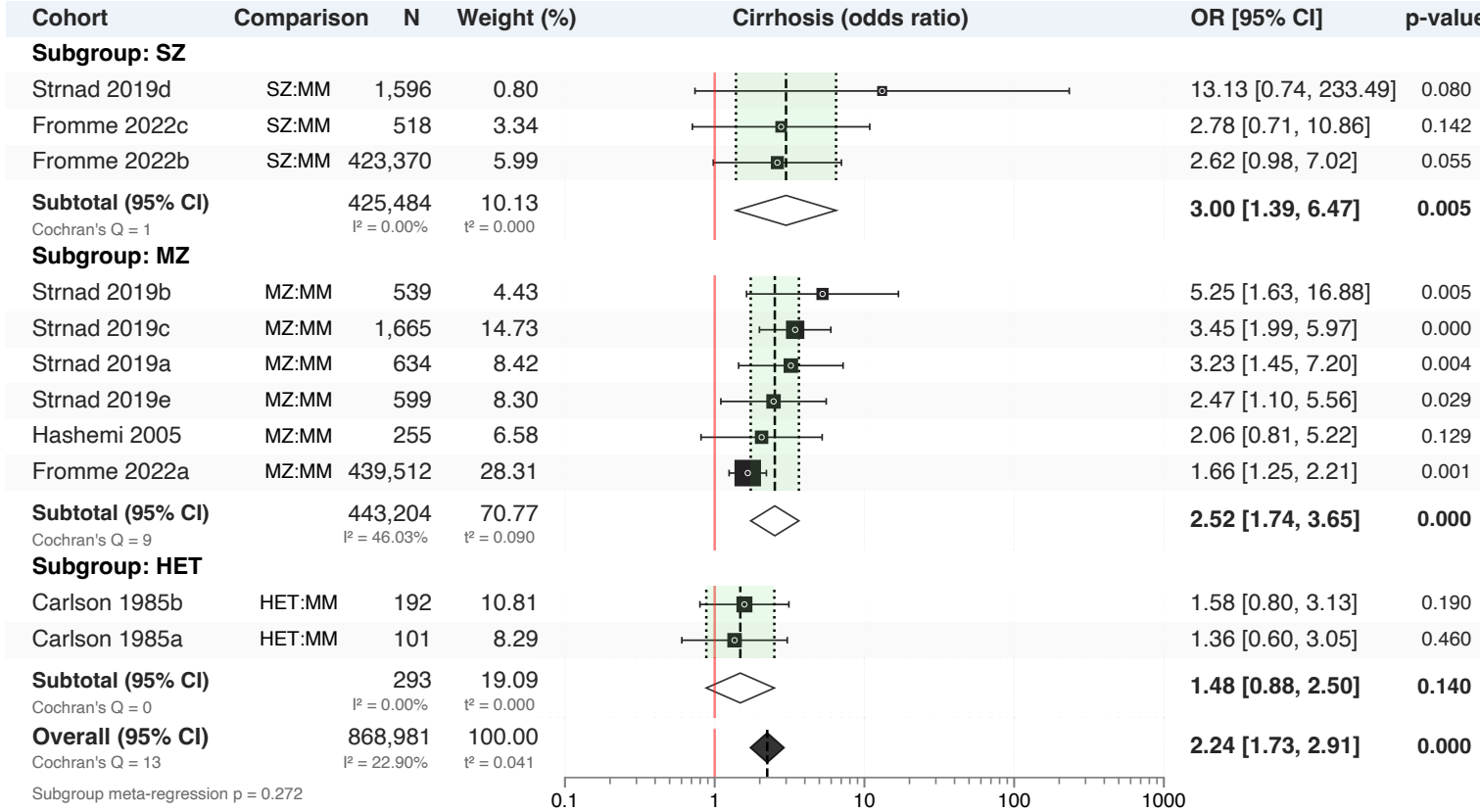

D

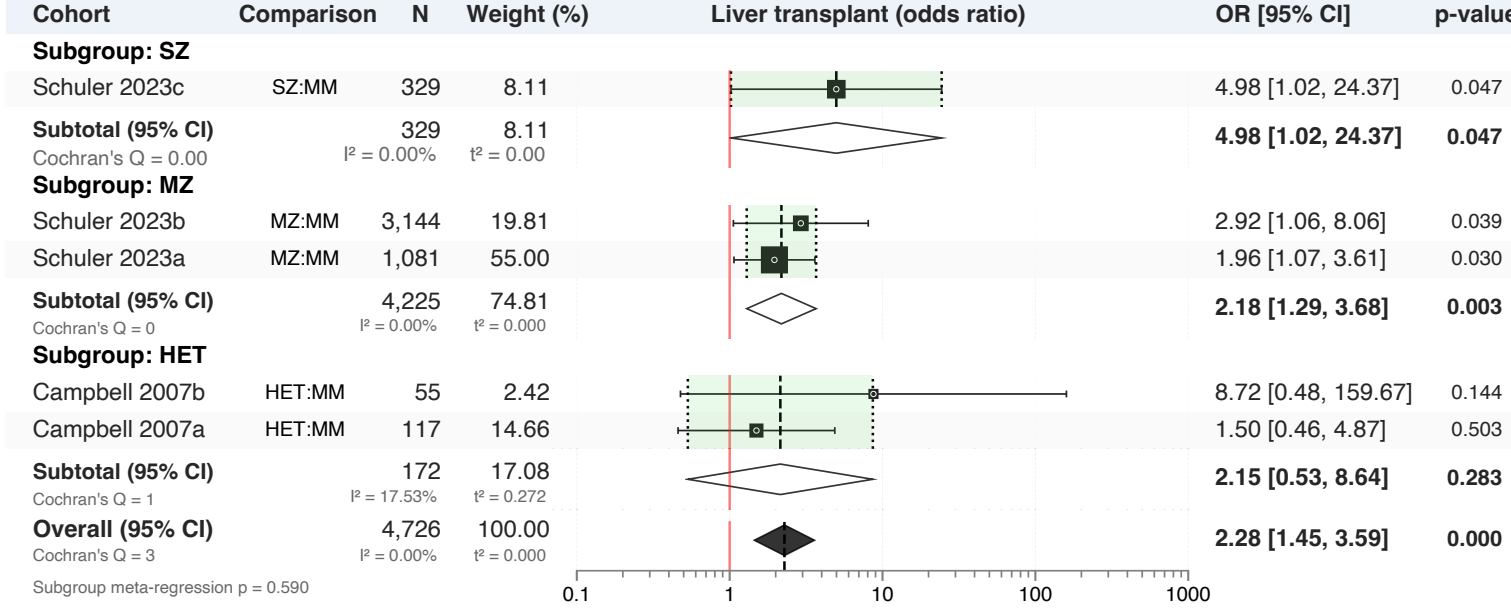

Supplement: Supplementary file 1 — Table S1: Search terms used in the systematic review. Table S2: Characteristics of included studies. Table S3: PRISMA checklist. Figure S1: Study selection and characteristics of included cohorts. (A) PRISMA flow diagram summarizing study identification, screening, eligibility assessment, and inclusion. (B) Cumulative number of eligible publications by year. (C) Sex distribution across SERPINA1 genotype groups, with random‐effects meta‐regression showing no significant association between female proportion and genotype. (D) Geographic distribution of included cohorts by country or region. Figure S2: Study quality assessment using the Newcastle–Ottawa Scale. Non‐randomized studies were scored from 0 to 9 across the domains of selection, comparability, and outcome. Total scores were categorized as high (7–9), fair (4–6), or low (0–3) quality. Figure S3: Leave‐one‐out sensitivity analysis. Panels show the impact of excluding individual studies on pooled estimates for (A) comorbidities associated with metabolic syndrome (obesity, type 2 diabetes, steatosis), (B) serum liver enzymes (ALT, AST, ALP), and (C) liver disease outcomes (fibrosis, cirrhosis, liver transplantation). Each point represents the pooled estimate recalculated after omitting the indicated study. The green band represents the 95% CI of the complete meta‐analysis. Labels indicate whether the leave‐one‐out pooled estimate differed from the complete‐set pooled estimate using a two‐sided z test. Exclusion of any single study does not materially change the summary estimates for any outcome, ns = p > 0.05, * = p < 0.05. Figure S4: Sensitivity analysis of metabolic comorbidities and hepatic steatosis stratified by SERPINA1 genotype. Pooled estimates are presented by genotype subgroup for obesity prevalence (A), BMI mean difference versus MM controls (B), type 2 diabetes prevalence (C), steatosis prevalence (D), and steatosis odds ratio versus MM controls (E). Subgroup estimates are shown for MZ, SZ, and combin [file APT-64-430-s001.zip › apt70814-sup-0001-Supinfo01/Supplementary Figure S6.pdf]

Supplementary Figure S7

A

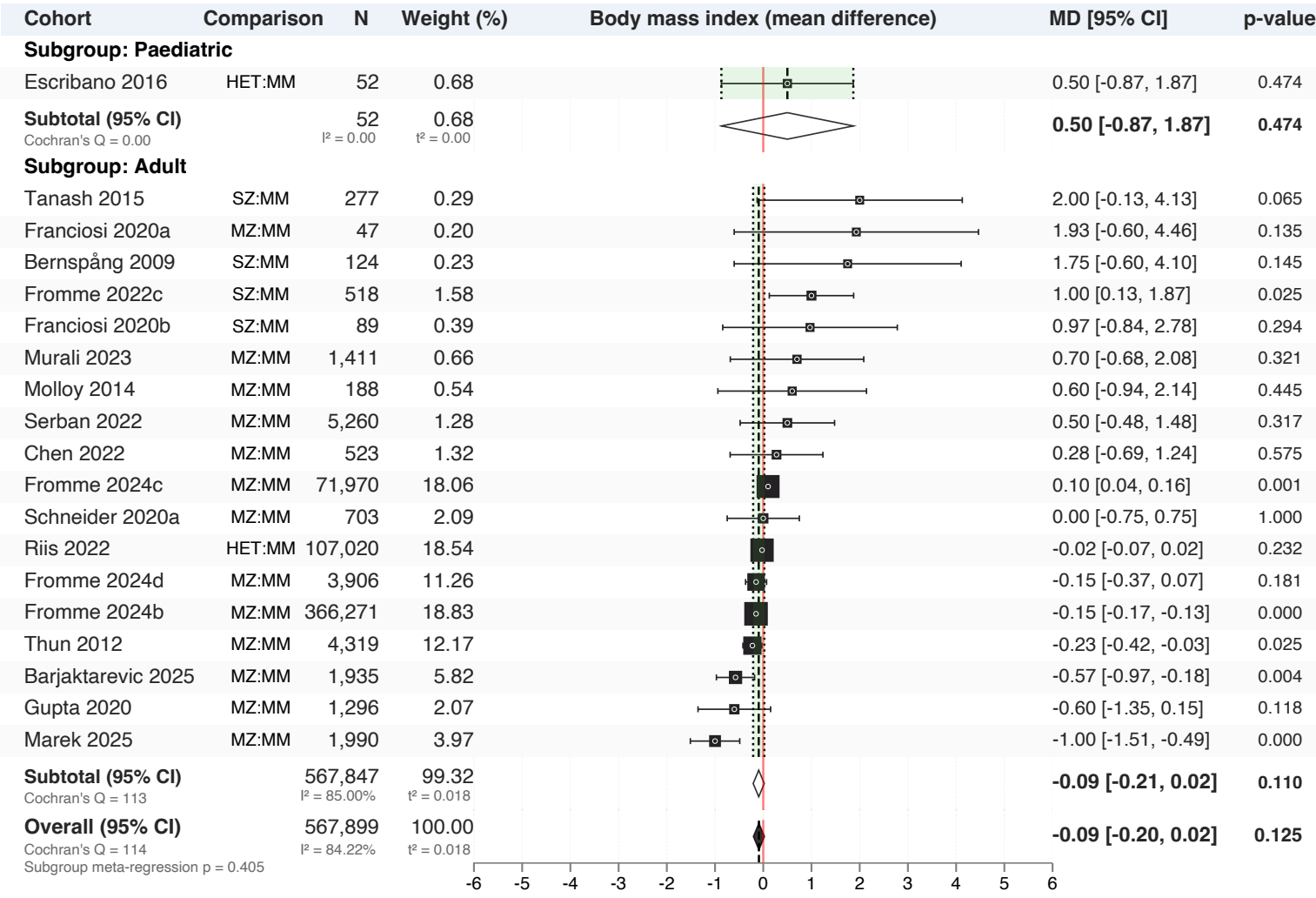

B

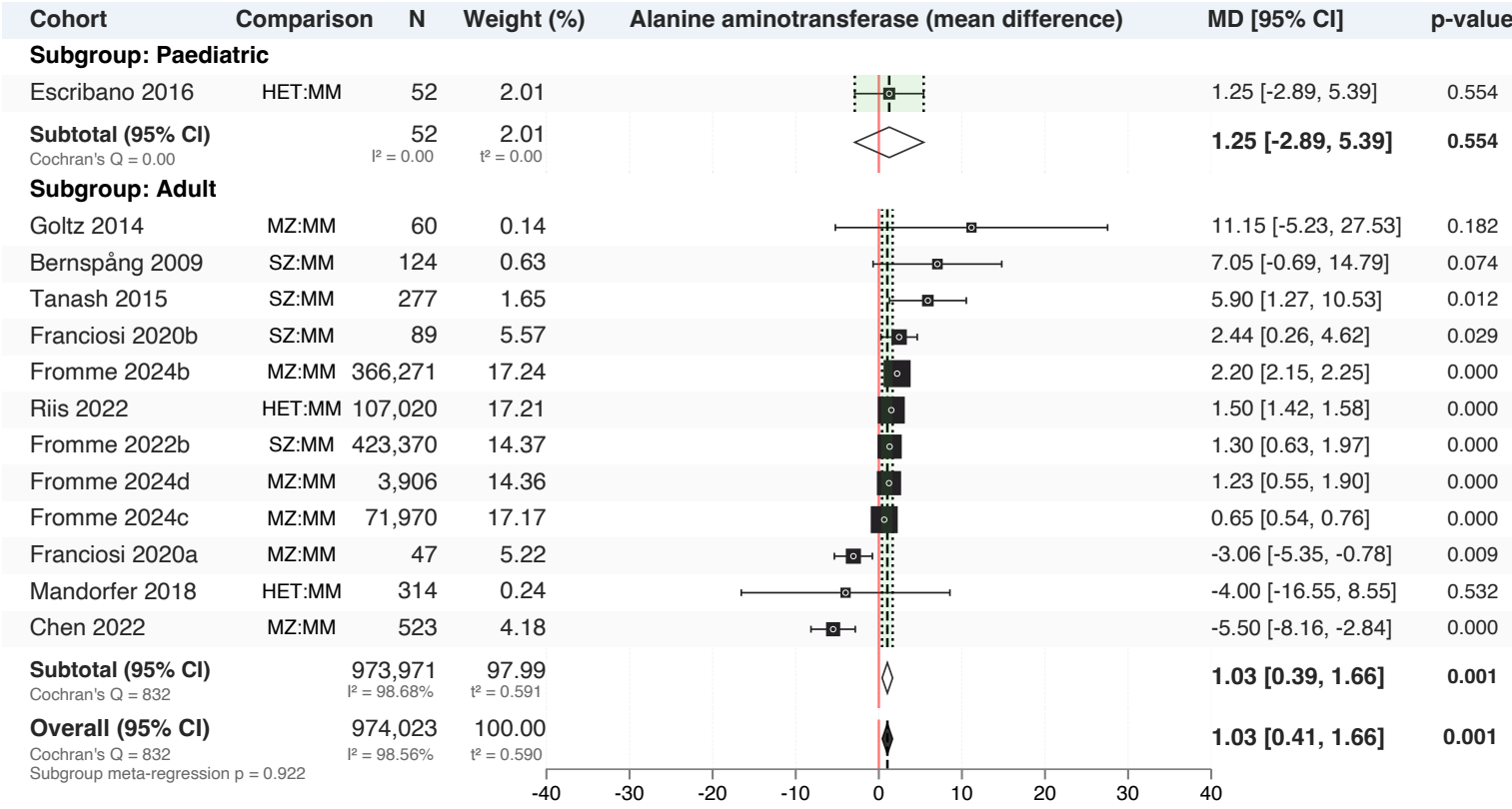

C

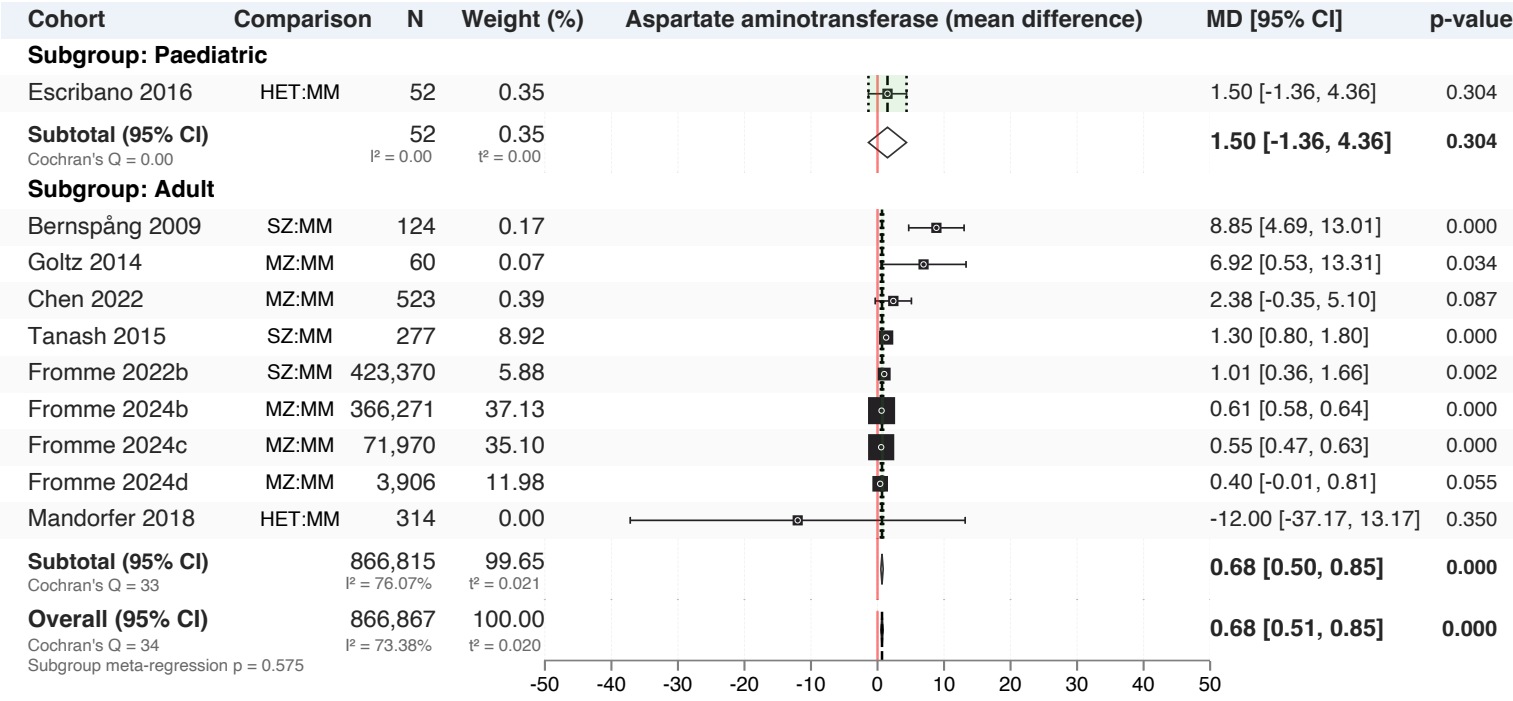

D

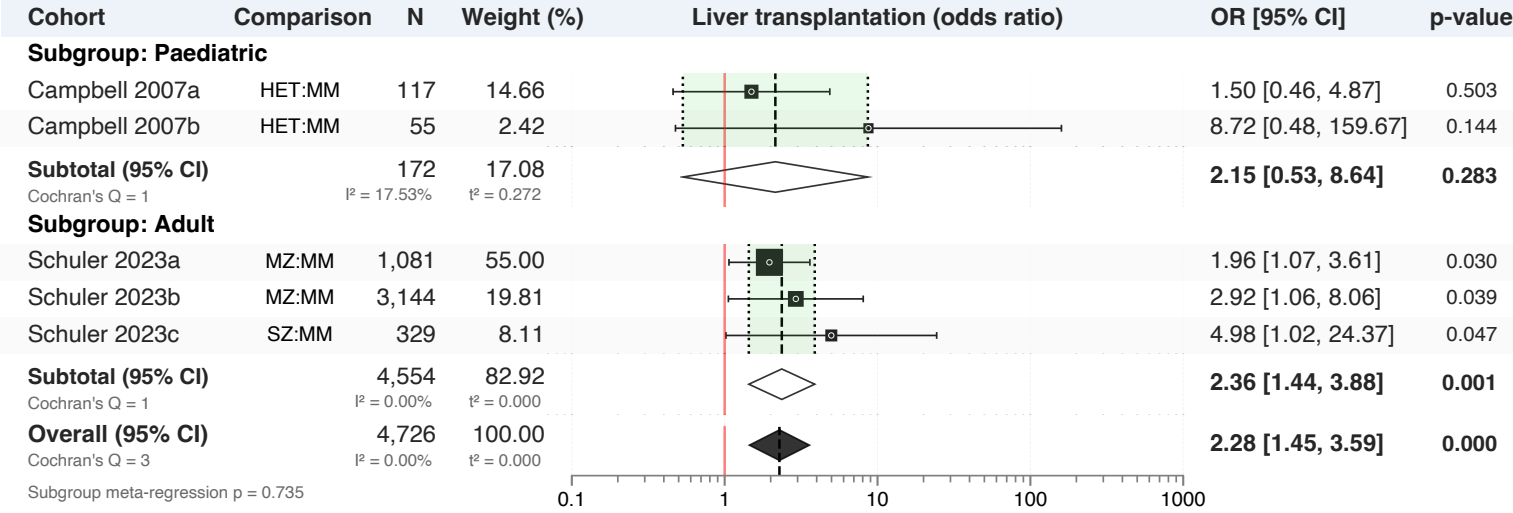

Supplement: Supplementary file 1 — Table S1: Search terms used in the systematic review. Table S2: Characteristics of included studies. Table S3: PRISMA checklist. Figure S1: Study selection and characteristics of included cohorts. (A) PRISMA flow diagram summarizing study identification, screening, eligibility assessment, and inclusion. (B) Cumulative number of eligible publications by year. (C) Sex distribution across SERPINA1 genotype groups, with random‐effects meta‐regression showing no significant association between female proportion and genotype. (D) Geographic distribution of included cohorts by country or region. Figure S2: Study quality assessment using the Newcastle–Ottawa Scale. Non‐randomized studies were scored from 0 to 9 across the domains of selection, comparability, and outcome. Total scores were categorized as high (7–9), fair (4–6), or low (0–3) quality. Figure S3: Leave‐one‐out sensitivity analysis. Panels show the impact of excluding individual studies on pooled estimates for (A) comorbidities associated with metabolic syndrome (obesity, type 2 diabetes, steatosis), (B) serum liver enzymes (ALT, AST, ALP), and (C) liver disease outcomes (fibrosis, cirrhosis, liver transplantation). Each point represents the pooled estimate recalculated after omitting the indicated study. The green band represents the 95% CI of the complete meta‐analysis. Labels indicate whether the leave‐one‐out pooled estimate differed from the complete‐set pooled estimate using a two‐sided z test. Exclusion of any single study does not materially change the summary estimates for any outcome, ns = p > 0.05, * = p < 0.05. Figure S4: Sensitivity analysis of metabolic comorbidities and hepatic steatosis stratified by SERPINA1 genotype. Pooled estimates are presented by genotype subgroup for obesity prevalence (A), BMI mean difference versus MM controls (B), type 2 diabetes prevalence (C), steatosis prevalence (D), and steatosis odds ratio versus MM controls (E). Subgroup estimates are shown for MZ, SZ, and combin [file APT-64-430-s001.zip › apt70814-sup-0001-Supinfo01/Supplementary Figure S7.pdf]

Supplementary Figure S8

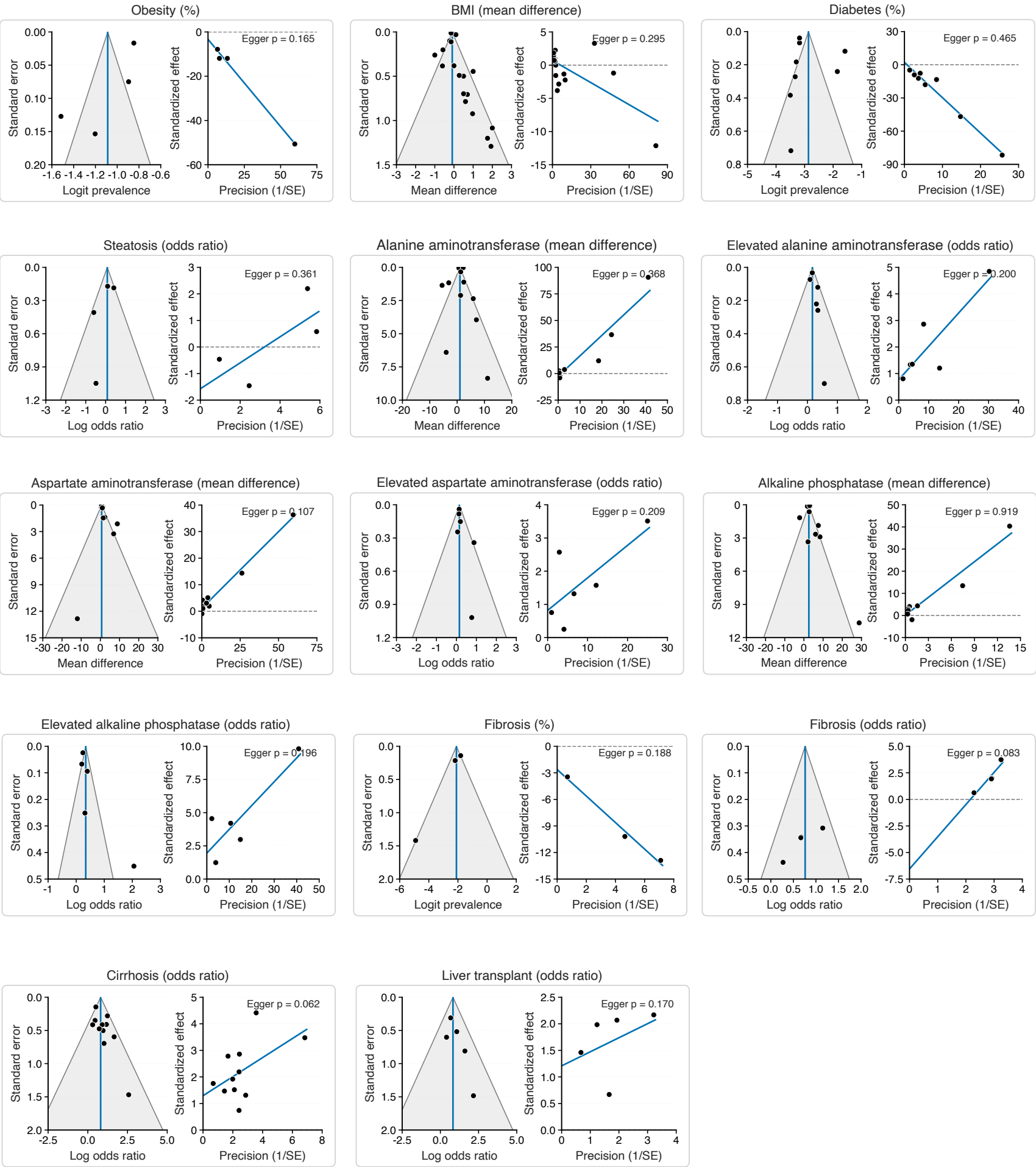

Supplement: Supplementary file 1 — Table S1: Search terms used in the systematic review. Table S2: Characteristics of included studies. Table S3: PRISMA checklist. Figure S1: Study selection and characteristics of included cohorts. (A) PRISMA flow diagram summarizing study identification, screening, eligibility assessment, and inclusion. (B) Cumulative number of eligible publications by year. (C) Sex distribution across SERPINA1 genotype groups, with random‐effects meta‐regression showing no significant association between female proportion and genotype. (D) Geographic distribution of included cohorts by country or region. Figure S2: Study quality assessment using the Newcastle–Ottawa Scale. Non‐randomized studies were scored from 0 to 9 across the domains of selection, comparability, and outcome. Total scores were categorized as high (7–9), fair (4–6), or low (0–3) quality. Figure S3: Leave‐one‐out sensitivity analysis. Panels show the impact of excluding individual studies on pooled estimates for (A) comorbidities associated with metabolic syndrome (obesity, type 2 diabetes, steatosis), (B) serum liver enzymes (ALT, AST, ALP), and (C) liver disease outcomes (fibrosis, cirrhosis, liver transplantation). Each point represents the pooled estimate recalculated after omitting the indicated study. The green band represents the 95% CI of the complete meta‐analysis. Labels indicate whether the leave‐one‐out pooled estimate differed from the complete‐set pooled estimate using a two‐sided z test. Exclusion of any single study does not materially change the summary estimates for any outcome, ns = p > 0.05, * = p < 0.05. Figure S4: Sensitivity analysis of metabolic comorbidities and hepatic steatosis stratified by SERPINA1 genotype. Pooled estimates are presented by genotype subgroup for obesity prevalence (A), BMI mean difference versus MM controls (B), type 2 diabetes prevalence (C), steatosis prevalence (D), and steatosis odds ratio versus MM controls (E). Subgroup estimates are shown for MZ, SZ, and combin [file APT-64-430-s001.zip › apt70814-sup-0001-Supinfo01/Supplementary Figure S8.pdf]
